# Positive surface charge of GluN1 N-terminus mediates the direct interaction with EphB2 and NMDAR mobility

**DOI:** 10.1038/s41467-020-14345-6

**Published:** 2020-01-29

**Authors:** Halley R. Washburn, Nan L. Xia, Wei Zhou, Yu-Ting Mao, Matthew B. Dalva

**Affiliations:** 1 Department of Neuroscience and Jefferson Center for Synaptic Biology, Thomas Jefferson University, 233 South 10th Street, Bluemle Life Sciences Building, Room 324, Philadelphia, PA 19107 USA; 2 Present Address: Department of Neurobiology, University of Chicago, 924 East 57th street, JFKR212, Chicago, IL 60637 USA

**Keywords:** Biophysics, Neuroscience, Synaptic development, Ion channels in the nervous system, Molecular neuroscience

## Abstract

Localization of the N-methyl-D-aspartate type glutamate receptor (NMDAR) to dendritic spines is essential for excitatory synaptic transmission and plasticity. Rather than remaining trapped at synaptic sites, NMDA receptors undergo constant cycling into and out of the postsynaptic density. Receptor movement is constrained by protein-protein interactions with both the intracellular and extracellular domains of the NMDAR. The role of extracellular interactions on the mobility of the NMDAR is poorly understood. Here we demonstrate that the positive surface charge of the hinge region of the N-terminal domain in the GluN1 subunit of the NMDAR is required to maintain NMDARs at dendritic spine synapses and mediates the direct extracellular interaction with a negatively charged phospho-tyrosine on the receptor tyrosine kinase EphB2. Loss of the EphB-NMDAR interaction by either mutating GluN1 or knocking down endogenous EphB2 increases NMDAR mobility. These findings begin to define a mechanism for extracellular interactions mediated by charged domains.

## Abstract (web summary)

NMDA receptors undergo constant cycling into and out of the postsynaptic density. Here authors show that NMDAR's GluN1 subunit is required to maintain NMDARs at dendritic spine synapses by direct extracellular interaction with the receptor tyrosine kinase EphB2.

## Introduction

Synaptic function and synaptic plasticity rely on the precise localization of glutamate receptors within the postsynaptic density of spine synapses^1–3^. N-methyl-d-aspartate receptors (NMDARs) are one of the three major types of ionotropic glutamate receptors and are essential for many types of plasticity^4^. NMDARs are heterotetramers composed of homologous subunits: two GluN1 and two GluN2 (four subtypes, A–D) or GluN3 (two subtypes, A and B). The extracellular domains of the GluN2 subunits contain the glutamate-binding site while GluN1 subunits bind co-agonist glycine and are essential for NMDAR function^4^. The extracellular region of the GluN1 subunit consists of two large domains, the ligand-binding domain (LBD), which contains the glycine-binding site, and the N-terminal domain (NTD). The clamshell-like structure of NMDAR subunit NTDs allosterically regulates NMDAR channel function by binding to modulators, such as zinc, polyamines, and ifenprodil^5,6^. While the molecules that bind to the GluN2B NTD and the GluN1/GluN2B NTD interface are well characterized^7–10^, binding partners for the GluN1 NTD remain unknown.

Retention of the NMDAR in the postsynaptic density is essential for synaptic plasticity. However, rather than remaining static at synaptic sites, synaptic NMDARs are in a dynamic equilibrium between diffusion-driven exchange with other membrane compartments and confinement at synaptic sites by protein–protein interactions^11^. Retention of NMDARs at synapses is regulated by both intracellular scaffolding proteins, such as PSD-95^12^, and extracellular interacting proteins, such as the EphB receptor tyrosine kinases (RTKs)^13^. While the impact of intracellular interactions on NMDAR mobility are well understood^14–16^, the role of extracellular interactions on the mobility of the NMDAR at synaptic sites is less well defined.

Ligand-dependent activation of EphB RTKs results in the direct interaction between the extracellular domains of EphB2 and the NMDAR GluN1 subunit^13^. Expression of EphBs is required for normal synaptic levels of NMDARs in the mature brain^17,18^. Interaction with EphB2 results in increased NMDAR-dependent calcium influx, modulation of NMDAR channel function, phosphorylation of the GluN2B subunit of the NMDAR, and enhanced surface retention of the NMDAR^13,17,19^. Moreover, defects in the EphB–NMDAR interaction are associated with multiple diseases including anti-NMDAR encephalitis and Alzheimer’s disease^20,21^. Binding of EphB2 to the NMDAR requires phosphorylation of Y504, an extracellular tyrosine (p*Tyr) residue in the fibronectin type-III (FN3) domain of EphB2. Phosphorylation of EphB2 Y504 is necessary and sufficient for the EphB–NMDAR interaction and a negative charge at Y504 is required for the EphB–NMDAR interaction and synaptic localization of the NMDAR^19^. However, the interaction site on GluN1 and the mechanism responsible for the EphB–NMDAR interaction are unknown.

Intracellular interactions between p*Tyr residues are often mediated by SH2 domain binding. SH2 domains mediate protein–protein interactions by recognizing specific p*Tyr residues within specific short linear amino acid sequences^22^. Among a number of factors^23,24^, one key determinant for recognition is the positive surface charge in regions of the SH2 domain that coordinates the negative charge of the p*Tyr residue^24,25^. However, whether similar mechanisms might regulate interactions between the extracellular domains of proteins is not known. The recent discovery of mechanisms which mediate extracellular phosphorylation^19,26–30^ and the requirement of a negatively charged p*Tyr for the EphB2–NMDAR interaction suggest that the interaction domain on the NMDAR responsible for the EphB–NMDAR interaction might be positively charged.

Here we use a combined approach of modeling and site-directed mutagenesis to define the region of the NMDAR that is required for the extracellular interaction with EphB2. We define an area within the hinge region of the GluN1 NTD as necessary for the EphB2–NMDAR interaction. Interestingly, the interaction domain emerges from the tertiary structure of the GluN1 subunit. Mutations in the hinge region that neutralize or reverse the positive surface potential reduce or eliminate the EphB2–NMDAR interaction. Disrupting the ability of GluN1 to interact with EphB2 increases the mobility of the NMDAR at dendritic spine synapses. These data define a charge-dependent mechanism mediating stabilization of the NMDAR in dendritic spines and suggest a non-canonical mechanism as a functional role for extracellular phosphorylation that may mediate protein–protein interactions.

## Results

### EphB2 interacts with NMDARs at synapses

**Fig. 1:**
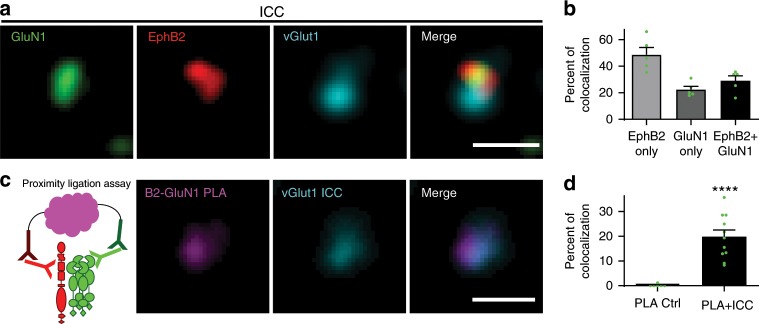
GluN1 interacts with EphB2 at synapses. **a** Representative images of synaptosomes immunostained for GluN1 in green, EphB2 in red, and vGlut1 in cyan. Far right panel shows merged image. Scale bar = 1.5 µm. **b** Quantification of the percentage of vGlut1^+^ synaptosomes that colocalize with either EphB2 only, GluN1 only, or both EphB2 and GluN1 (green dots represent *n* = 612 synaptosomes, five fields). **c** Schematic of proximity ligation assay shows EphB2 in red and NMDAR in green. Rolling circle amplification of fluorescently tagged oligonucleotides (represented in magenta) occurs after primary and secondary probes bind. Representative images of synaptosomes immunostained for vGlut1 in cyan (middle panel). PLA between EphB2 and GluN1 is shown in magenta (left panel). Right panel shows merged image. Scale bar = 1.5 µm. **d** Quantification of the percentage of vGlut1^+^ synaptosomes that colocalize with PLA puncta (PLA + ICC). The control condition (PLA Ctrl) was performed without EphB2 primary antibody (****p < 0.0001, unpaired *t*-test; green dots represent PLA Ctrl *n* = 569, 6 fields; PLA + ICC *n* = 421 synaptosomes, 11 fields).

EphB2 and the NMDAR interact in brain lysates, at synapses in cultured neurons, and directly in vitro^17,19^. To understand the mechanism underlying the EphB–NMDAR interaction, we tested whether endogenous EphB2 and NMDARs interact at synapses using synaptosomes isolated from mouse brain (Supplementary Fig. 1a)^19,31,32^. Synaptosomes provide a system in which epitopes for antibody binding are accessible while maintaining much of the endogenous synaptic organization^32,33^. Immunostaining of synaptosomes for endogenous EphB2 and GluN1 reveals that these proteins are found at 77.6% (EphB2) and 51.4% (GluN1) of vGlut1-positive sites (Fig. 1a, b, Supplementary Fig. 1b). Approximately 30% of vGlut1-positive synaptosomes colocalized with both EphB2 and GluN1 (29 ± 3.7%; Fig. 1a, b, Supplementary Fig. 1b). To test whether these proteins might be interacting, we conducted immunocytochemistry (ICC) for vGlut1-positive presynaptic sites followed by rolling-circle amplification proximity ligation assays (PLAs) in synaptosomes with antibodies against the extracellular domains of GluN1 (anti-GluN1) and EphB2 (anti-EphB2; PLA schematic; Fig. 1c, Supplementary Fig. 1c)^34^. Consistent with our immunostaining data, ~20% of vGlut1-positive sites showed endogenous EphB2–GluN1 PLA signal (19.8 ± 2.7%; Fig. 1d, Supplementary Fig. 1c). These findings suggest that EphB2 and GluN1 interact at most synaptic sites where both proteins colocalize.

### The NTD of GluN1 is necessary for the EphB–NMDAR interaction

NMDA receptor encephalitis is a rare autoimmune disease characterized by auto-antibody-binding to the NMDAR NTD^35^. Patient-derived anti-NMDAR antibodies disrupt the EphB–NMDAR interaction, decreasing surface expression and increasing NMDAR mobility^20^. Conversely, treatment of neurons with the EphB ligand, ephrin-B2, induces EphB to compete with patient auto-antibodies for NMDAR binding, increasing the EphB–NMDAR interaction and blocking the effects of the patient-derived antibodies^20,36^. These data suggest that the GluN1 NTD hinge region might be important for the EphB–NMDAR interaction^35^. Therefore, we sought regions of the GluN1 extracellular domain near the NTD hinge with positive surface potential that might interact with the negatively charged EphB2 p*Y504 site.

**Fig. 2:**
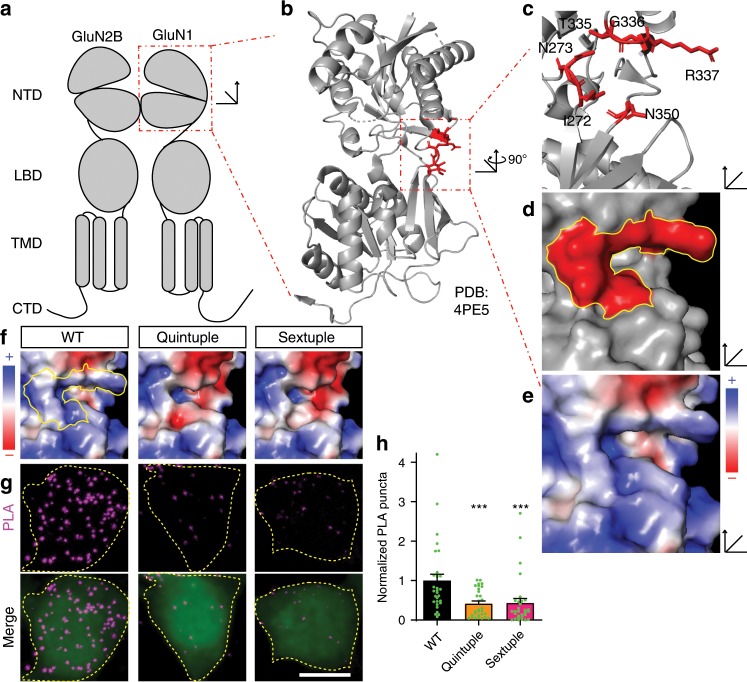
GluN1 hinge mutants disrupt the EphB2–GluN1 interaction in HEK293T cells. **a** A model of domain organization of the GluN1 and the GluN2B subunits of the NMDA receptor. The red box indicates the N-terminal domain (NTD) of GluN1. **b** The crystal structure of the NTD of GluN1 (PDB: 4PE5). The red box indicates the hinge region within the NTD with specific amino acids highlighted in red. **c** The structure of the GluN1 NTD hinge region represented in cartoon form with the location of six amino acids highlighted in red (I272, N273, T335, G336, R337, N350). **d** The surface representation of the structure of the GluN1 NTD hinge region with the location of the six amino acids in **c** highlighted in red. **e** Charge map of the GluN1 NTD hinge region. The charge map was generated using the adaptive Poisson–Boltzmann solver (APBS) plugin in PyMOL. **f** Charge maps of GluN1 NTD hinge region mutants (WT, Quintuple, Sextuple). Yellow outline indicates the location of the six key hinge region amino acids in WT GluN1. **g** Representative images of the PLA assay results in HEK293T cells. HEK293T cells were transfected with either WT Myc-GluN1, Quintuple mutant Myc-GluN1 (I272A/N273A/T335A/G336A/R337A), or Sextuple mutant Myc-GluN1 (I272A/N273A/T335A/G336A/R337A/350Q), and GluN2B, FLAG-tagged-EphB2, and EGFP. The upper panels show PLA signal alone. The lower panels are the merged images with EGFP in green and PLA signal in magenta. Scale bar = 10 µm. **h** Quantification of the effects of GluN1 mutants on PLA puncta number. PLA puncta number are quantified by counting the number of puncta per 100 µm^2^ in EGFP^+^ cells and normalizing to the WT condition (***p < 0.005, ANOVA; green dots represent *n* = 30 cells for each condition).

To determine the surface potential within the NTD of GluN1 (Fig. 2a), we used the crystal structure of the GluN1 NTD (PDB: 4PE5; Fig. 2b) and the adaptive Poisson–Boltzmann solver (APBS) to visualize the electrostatic surface potential^37^. The hinge region is positively charged on the exposed surface of the protein, facing away from the channel and away from the GluN2B subunit (Fig. 2a, e, positive = blue, negative = red, neutral = white). In contrast, the homologous region of GluN2B (PDB: 4PE5) is negatively charged (Supplementary Fig. 2a). The region responsible for the positive surface potential within the GluN1 NTD is composed of six amino acids (I272, N273, T335, G336, R337, and N350) (Fig. 2c, d). Interestingly, unlike canonical SH2 domain tyrosine-binding domains^24,38^, the residues generating the positively charged region in the NTD are not in β-sheets or a linear sequence, but instead are in unstructured loops in spatial proximity to one another.

To begin to determine whether the positive surface charge of the NTD hinge region might play a role in the EphB–NMDAR interaction, all six amino acids forming the positive region of the NTD hinge were mutated (Sextuple GluN1 mutant: I272A/N273A/T335A/G336A/R337A/N350Q). APBS predicts that the Sextuple mutant will result in a negative surface potential compared to wild-type (WT) GluN1 (Fig. 2f, right). One of the amino acids identified, N350, is an N-linked glycosylation site^39^. Consistent with these findings, the GluN1 Sextuple mutant with N350Q migrates on a western blot with a shift in apparent molecular weight consistent with a loss of glycosylation (Supplementary Fig. 2b). Therefore, although glycosylation of N350 does not appear to regulate surface localization of the NMDAR^39^, we also generated a Quintuple GluN1 mutant that lacks the mutation at N350 (Quintuple GluN1 mutant: I272A/N273A/T335A/G336A/R337A). APBS indicates that the Quintuple mutant also results in a significant change in the surface potential in the hinge region (Fig. 2f).

To test whether the GluN1 hinge region charge mutants disrupt the EphB2–NMDAR interaction, we  performed PLA on unpermeablized HEK293T cells expressing EGFP, FLAG-tagged EphB2 (FLAG-EphB2), GluN2B, and either WT, Quintuple, or Sextuple mutant Myc-tagged GluN1 (Myc-GluN1). The epitope tags on both GluN1 and EphB2 were placed in the extracellular domains after the signal peptide. Control experiments validated that PLA puncta are specific to the presence of both proteins and both antibodies (Supplementary Fig. 1d, e), showed that these proteins were on the cell surface (Supplementary Fig. 2c, d), and that GluN1 mutants expressed at similar or slightly higher levels compared to WT GluN1 in HEK293T cells (Supplementary Fig. 2e–g). These data suggest that any reductions in the EphB2–NMDAR interaction detected are not due to decreases in protein expression or failure of the GluN1 subunits to reach the cell surface.

To determine whether the surface charge of the GluN1 NTD hinge region is important for the EphB–NMDAR interaction, transfected HEK293T cells were fixed and PLA was performed for the extracellular domains of GluN1 (anti-Myc) and EphB2 (anti-EphB2)^34^. The interaction between GluN1 and EphB2 was determined by quantifying the number of PLA puncta in EGFP-positive cells^40,41^. The EphB2–NMDAR interaction was significantly reduced in both Quintuple and Sextuple mutant GluN1-transfected conditions compared to WT (Fig. 2g, h, ***p < 0.005, ANOVA). These data suggest that a positive surface potential in the hinge region of the GluN1 NTD is necessary for the EphB–NMDAR interaction.

We next asked whether changing the surface potential of the GluN1 NTD hinge region might disrupt the EphB2–NMDAR interaction in neurons. Experiments in primary cortical rat neurons were conducted using a molecular replacement strategy where endogenous GluN1 was knocked out using transfectable CRISPR reagents (Supplementary Fig. 3)^42,43^. Neurons were transfected with WT, Quintuple mutant, or Sextuple mutant EGFP–GluN1, as well as GluN2B and mCherry at day in vitro 3 (DIV3). Both mutant and WT GluN1 proteins were localized to the neuronal surface (Supplementary Fig. 4a, b) and all mutant receptor-channels fluxed calcium in response to glutamate application with kinetics similar to wild-type at DIV8-10 (Supplementary Fig. 5)^44^.

**Fig. 3:**
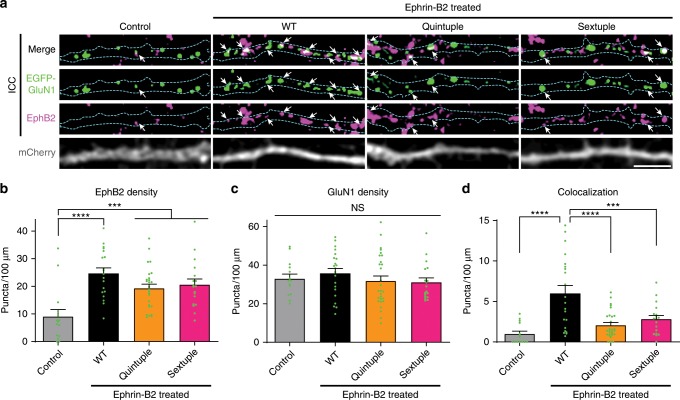
GluN1 hinge mutants disrupt the EphB2–GluN1 interaction in neurons. **a** Representative images of dendrites of DIV6-9 cortical neurons transfected with EGFP–GluN1 (WT, Quintuple, or Sextuple), mCherry, GluN2B, and CRISPR constructs to knock out endogenous GluN1. The control condition is WT GluN1-transfected treated with control reagents instead of ephrin-B2. The other three conditions were treated with ephrin-B2 for 45 min to induce the interaction between the NMDAR and EphBs. Top row panels show the colocalization between EGFP–GluN1 and EphB2 in white. Second row panels show the high contrasted images of EGFP–GluN1 puncta in green. Third row panels show the high contrasted images of endogenous EphB2 in magenta. Last row panels show mCherry. Outlines of morphology are shown in cyan. Scale bar = 5 µm. **b** Quantification of EphB2 density (puncta number per 100 µm) in indicated groups (***p < 0.005, ****p < 0.0001, ANOVA followed by Tukey’s; green dots represent Control *n* = 14; WT *n* = 21 cells; Quintuple *n* = 28; Sextuple *n* = 18). **c** Quantification of EGFP–GluN1 density (puncta number per 100 µm) in indicated groups (*p* = 0.5958, ANOVA; green dots represent Control *n* = 14; WT *n* = 21 cells; Quintuple *n* = 28; Sextuple *n* = 18). **d** Quantification of the effects of the different GluN1 mutants on colocalization (puncta number per 100 µm) between GluN1 and EphB2 (***p < 0.005, ****p < 0.0001, ANOVA; green dots represent Control *n* = 14; WT *n* = 21 cells; Quintuple *n* = 28; Sextuple *n* = 18).

Next, we asked whether mutations to the hinge region of the GluN1 NTD might disrupt the EphB2–NMDAR interaction. To test this, we used a previously described assay in which DIV6-9 neurons are treated with soluble activated ephrin-B2 for 45 min to induce the surface interaction between EphB2 and the NMDAR^13,17,19^. The colocalization of transfected EGFP–GluN1 and endogenous EphB2 was quantified using custom macros^19,45^. Ephrin-B2 activation resulted in clustering of EphB2 in all conditions (Fig. 3a, b). GluN1 and EphB2 puncta density were the same across the three ephrin-treated groups (Fig. 3b, *p* = 0.1006; Fig. 3c, *p* = 0.4199; ANOVA), suggesting that effects on colocalization are not due to changes in EphB2 or NMDAR expression or puncta number. Ephrin treatment of WT GluN1-transfected neurons resulted in increased EphB2–GluN1 co-clustering compared to control (Fig. 3a, d, ****p < 0.0001, ANOVA). Both the Quintuple and Sextuple GluN1 hinge mutants were significantly less well colocalized with EphB2 than WT GluN1 after ephrin treatment (Fig. 3a, d, ****p < 0.0001, ***p < 0.005; ANOVA). Together, these data suggest that the positive surface charge of the hinge region of the NTD is necessary for the EphB–NMDAR interaction.

### Surface potential of the GluN1 hinge mediates NMDAR mobility

The EphB–NMDAR interaction regulates the stability of the NMDAR at synaptic sites in mature neurons^17^. Mice lacking EphB1–3 have reduced levels of NMDARs at synaptic sites and knockdown of EphB2 in mature neurons drives GluN2B-containing NMDARs from synapses^17^. Consistent with the model that these events depend on the EphB–NMDAR interaction, treatment of neurons with patient-derived anti-NMDAR antibodies that block the EphB–NMDAR interaction increases the diffusion of NMDARs out of synapses^20^. These data suggest that the EphB–NMDAR interaction is important for the synaptic stability of the NMDAR.

**Fig. 4:**
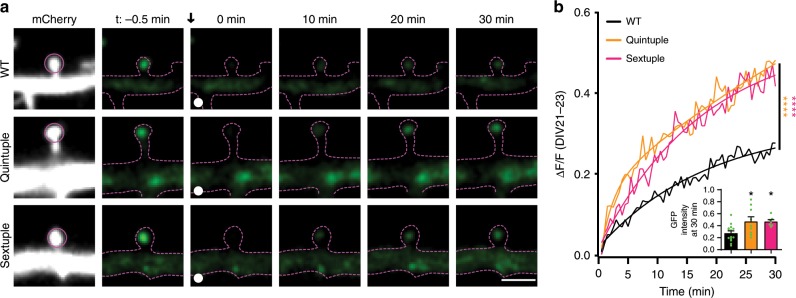
Surface charge of the GluN1 NTD hinge region affects NMDAR mobility in mature neurons. **a** Representative FRAP images at different time points of DIV21–23 cortical neurons transfected with EGFP–GluN1 (WT, Quintuple, or Sextuple). Recovery of bleached spine puncta (magenta circle) was monitored for 30 min at 30-s intervals. Scale bar = 2 µm. **b** Quantification of the recovery curve of different GluN1 mutants in DIV21–23 cortical neurons. Graphs represent mean intensity and show fit (****p < 0.0001, Kolmogorov–Smirnov (KS) nonparametric test). Inset: Quantification of the mobile fraction of EGFP–GluN1 puncta in dendritic spines at 30 min after  photobleaching in EGFP–GluN1 mutant transfected cells compared to WT EGFP–GluN1 in DIV21–23 (*p < 0.05, ANOVA; green dots represent WT *n* = 14 puncta; Quintuple *n* = 11; Sextuple *n* = 10). Error bars show S.E.M.

We next asked whether decreasing the positive surface potential of the hinge region of the NTD, which reduces the EphB–NMDAR interaction, might increase mobility of the NMDAR at synaptic sites. We focused on older neurons, as the EphB–NMDAR interaction regulates the localization of the NMDAR in mature neurons^17,46^. Neurons were transfected with WT, Quintuple, or Sextuple EGFP–GluN1, GluN2B, mCherry, and CRISPR constructs to knockout endogenous GluN1^42^ at DIV14. The mobility of the NMDAR in mature neurons (DIV21–23) was measured using fluorescence recovery after photobleaching (FRAP) in mCherry-positive EGFP–GluN1-expressing neurons. The relative intensity of the diffuse and clustered EGFP–GluN1 signal was not significantly different across groups, suggesting that overall localization of mutants was similar to WT (Supplementary Fig. 4c, d). Over 80% of GluN1 puncta localized to the cell surface and only EGFP–GluN1 puncta in dendritic spines were selected for photobleaching to ensure our results were not reflective of the intracellular pool of receptors (Supplementary Fig. 4a, b, Fig. 3a). FRAP of EGFP–GluN1 puncta was determined by bleaching and then monitoring recovery of a puncta for 30 min, imaging once every 30 s. Both GluN1 hinge mutants recovered at a significantly higher rate than the WT (Fig. 4b, *p* < 0.001, Kolmogorov–Smirnov (KS) nonparametric test) and to a higher degree than WT EGFP–GluN1 (Fig. 4b inset, *p < 0.05, ANOVA). These data indicate that the mutant NMDARs with less positive surface charge have a larger mobile fraction compared to WT, likely resulting from the disruption of the interaction between EphB and the NMDAR.

### Impact of glycosylation of GluN1 N350 in HEK293T cells

Within the positive surface potential of the NTD hinge region, N350 is an N-linked glycosylation site^39,47^. To discern whether the glycosylation or the charge of N350 is more important for GluN1’s interaction with EphB2, we made two mutants: GluN1 N350Q and GluN1 N350A. Both mutations to N350 likely result in a loss of glycosylation as seen by the shift in western blot migration (Supplementary Fig. 6a), however APBS predicts that the N350Q mutation will also drive the surface potential of the NTD hinge region negative, while the N350A mutation will result in little detectable change in surface potential (Supplementary Fig. 6e). To test whether N350 is necessary for the EphB–NMDAR interaction, we conducted PLA in HEK293T cells transfected with FLAG-EphB2, EGFP, and either WT, N350Q or N350A Myc-GluN1. Mutants expressed at similar or slightly higher levels compared to WT GluN1 in HEK293T cells (Supplementary Fig. 6b–d). GluN1 N350Q-transfected cells, but not N350A, had significantly fewer PLA puncta than WT GluN1-transfected cells (Supplementary Fig. 6f, g, WT vs. N350Q, ***p = 0.0018; WT vs. N350A, *p* = 0.1055; N350A vs. N350Q, *p* = 0.3078; ANOVA). These data suggest that the ability of the NMDAR to interact with EphB2 is independent of glycosylation state in the hinge region.

### The amino acids required for the EphB2–GluN1 interaction

**Fig. 5:**
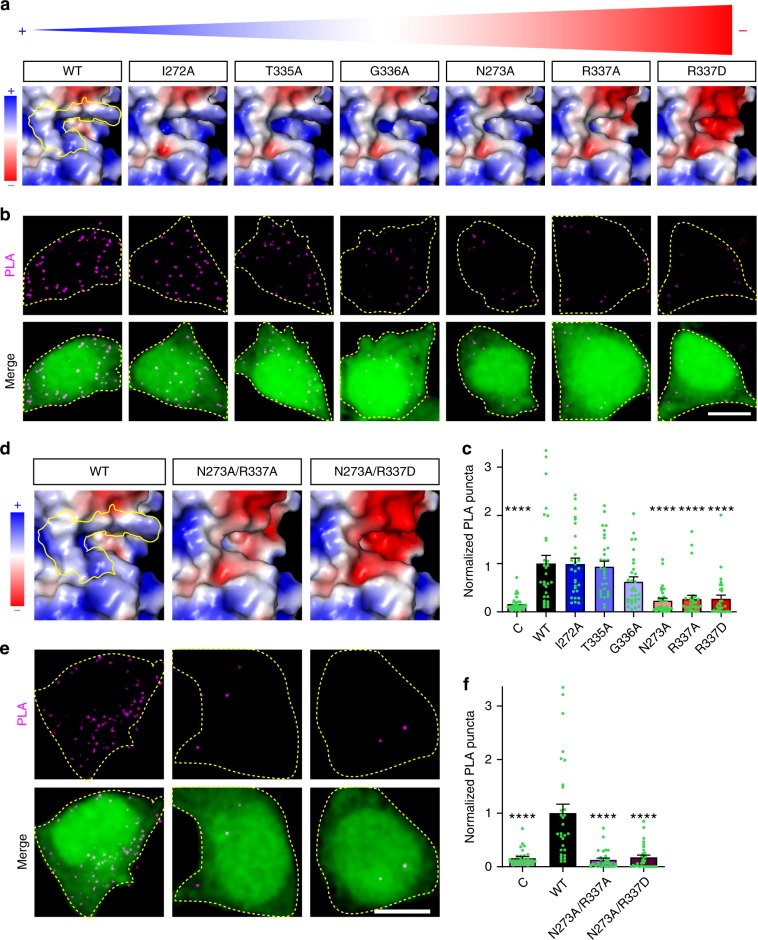
Specific hinge region amino acid residues are responsible for the EphB2–GluN1 interaction in HEK293T cells. **a** Surface charge maps of GluN1 NTD hinge region of the indicated GluN1 single point mutants in order of increasing negative charged in the hinge region (left (positive) to right (negative)), with blue representing positive charge and red representing negative charge. Yellow outline indicates the location of the six key hinge region amino acids in WT GluN1. **b** Representative images of PLA results in HEK293T cells. HEK293T cells were transfected with the indicated Myc-GluN1 single point mutants, together with GluN2B, FLAG-EphB2, and EGFP. The upper panels show PLA signal alone. The lower panels are merged images of EGFP in green and PLA signal in magenta. Scale bar = 10 µm. **c** Quantification of the effects of GluN1 mutants on PLA puncta number. PLA puncta number are quantified by counting the number of puncta per 100 µm^2^ in EGFP^+^ cells and normalizing to the WT condition (****p < 0.0001, ANOVA; green dots represent *n* = 30 cells for each condition). **d** Surface charge maps of GluN1 NTD hinge region of the indicated GluN1 double point mutants as in **a** (left (positive) to right (negative)). Yellow outline indicates the location of the six key hinge region amino acids in WT GluN1. **e** Representative images of PLA results in HEK293T cells. HEK293T cells were transfected with the indicated Myc-GluN1 double point mutants, together with GluN2B, FLAG-EphB2, and EGFP. The upper panels show PLA signal alone. The lower panels are merged images of EGFP in green and PLA signal in magenta. Scale bar = 10 µm. **f** Quantification of the effects of GluN1 mutants on PLA puncta number. PLA puncta number are quantified by counting the number of puncta per 100 µm^2^ in EGFP^+^ cells and normalizing to the WT condition (****p < 0.0001, ANOVA; green dots represent *n* = 30 cells for each condition).

To begin to determine which amino acids in the NTD hinge region might mediate the EphB–NMDAR interaction, we generated six individual  point mutants to each of the amino acids that form the area of positive surface charge (I272A, N273A, T335A, G336A, R337A, R337D). These mutants were chosen because they affect surface charge and not the glycosylation state of the GluN1 subunit. Each of these mutants trafficked to the cell surface at similar levels and ran as expected on western blots (Supplementary Fig. 7a–d, 7c, *p* = 0.0613; 7d, *p* = 0.1349; ANOVA). APBS models showed that mutations to N273 and R337 had the largest impact on the surface potential (N273A, R337A, and R337D) (Fig. 5a). If the positive surface charge of the GluN1 NTD hinge region mediates the EphB–NMDAR interaction, then the three GluN1 mutants that affect surface potential the most should disrupt the EphB2–NMDAR interaction. To test whether those charge mutants affect the interaction, PLA was performed on HEK293T cells transfected with each of the GluN1 single mutants (I272A, N273A, T335A, G336A, R337A, R337D), together with GluN2B, EphB2, and EGFP (Fig. 5b). Consistent with the charge-based model for the EphB–NMDAR interaction, the GluN1 mutants with the largest apparent effect on surface charge, N273A, R337A, and R337D, had significantly fewer PLA puncta per cell than WT (Fig. 5c, ****p < 0.0001, ANOVA). Mutations that did not alter the surface charge did not impact the number of PLA puncta (Fig. 5c, I272A vs. WT, *p* = 1.000; T335A vs. WT, *p* = 0.999; G336A vs. WT, *p* = 0.1489; ANOVA). Similarly, Myc-GluN1 N273 and R337 mutants co-immunoprecipitated FLAG-EphB2 less well than WT Myc-GluN1 (Supplementary Fig. 8). These data suggest that a positive surface potential in the NTD hinge region is necessary for the EphB–NMDAR interaction.

To test whether positive surface potential is important for the EphB–NMDAR interaction, we asked whether combining point mutants that affect the surface charge within the putative interaction domain might alter the EphB2–NMDAR interaction. To test this, we generated GluN1 double point mutants that APBS prediction indicated will have a negative surface potential (N273A/R337A and N273A/R337D; Fig. 5d). Both mutants express at similar levels, are found on the cell surface at similar levels, and migrate on western blots as expected (Supplementary Fig. 7e–h; 7g, *p* = 0.1921; 7h, *p* = 0.4097; ANOVA). As expected, PLA reveals that both double point mutants (N273A/R337A and N273A/R337D) interact significantly less well with FLAG-EphB2 compared to WT GluN1 in HEK293T cells (Fig. 5e, f, ****p < 0.0001, ANOVA). These data suggest that the positive surface charge generated by amino acids N273 and R337 of the NTD hinge region of the NMDAR GluN1 subunit is required for the EphB–NMDAR interaction.

### Disruption of the EphB2–NMDAR interaction

**Fig. 6:**
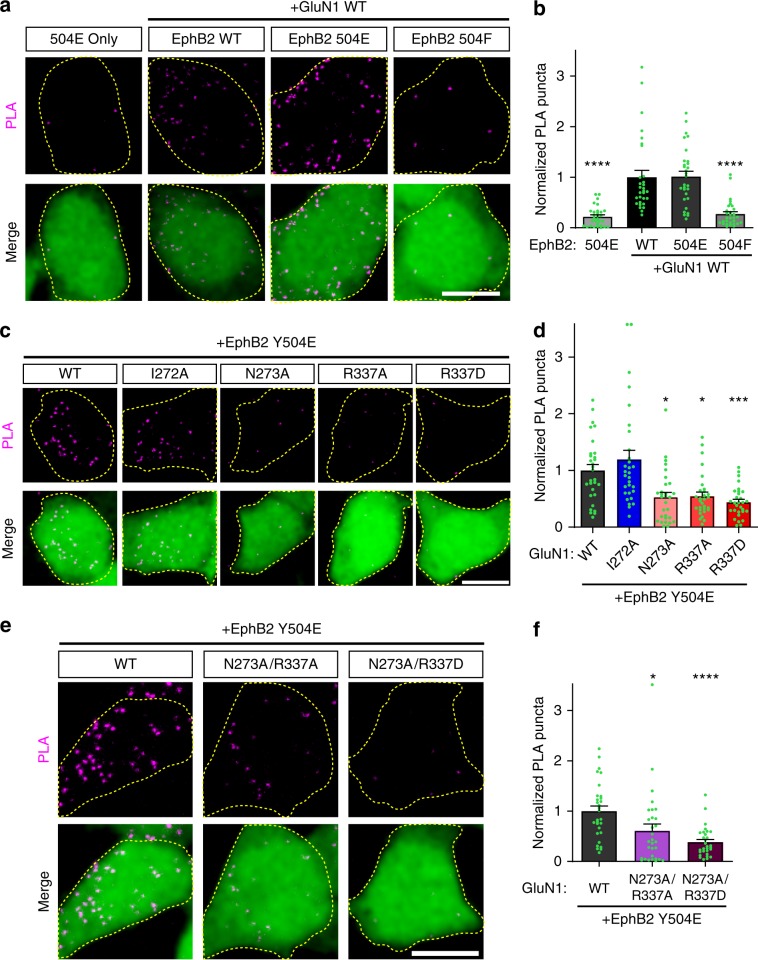
EphB2–GluN1 interaction in HEK293T cells is charge-dependent. **a** Representative images of PLA results in HEK293T cells. HEK293T cells were transfected with the indicated FLAG-EphB2 single point mutants (WT, Y504E, or Y504F), together with Myc-GluN1 WT, GluN2B, and EGFP. The upper panels show PLA signal alone. The lower panels are merged images of EGFP in green and PLA signal in magenta. Scale bar = 10 µm. **b** Quantification of the effects of EphB2 mutants on PLA puncta number. PLA puncta number are quantified by counting the number of puncta per 100 µm^2^ in EGFP^+^ cells and normalizing to the WT condition. (****p < 0.0001, ANOVA; *n* = 30 cells for each condition). **c** Representative images of PLA results in HEK293T cells. HEK293T cells were transfected with the indicated Myc-GluN1 single point mutants, together with GluN2B, FLAG-EphB2 Y504E, and EGFP. The upper panels show PLA signal alone. The lower panels are merged images of EGFP in green and PLA signal in magenta. Scale bar = 10 µm. **d** Quantification of the effects of GluN1 mutants on PLA puncta number. PLA puncta number are quantified by counting the number of puncta per 100 µm^2^ in EGFP^+^ cells and normalizing to the WT condition. (*p < 0.05, ***p < 0.005, ANOVA; *n* = 30 cells for each condition). **e** Representative images of PLA results in HEK293T cells. HEK293T cells were transfected with the indicated Myc-GluN1 double point mutants, together with GluN2B, FLAG-EphB2 Y504E, and EGFP. The upper panels show PLA signal alone. The lower panels are merged images of EGFP in green and PLA signal in magenta. Scale bar = 10 µm. **f** Quantification of the effects of GluN1 mutants on PLA puncta number (*p = 0.0219, ****p < 0.0001, ANOVA; green dots represent *n* = 30 cells for each condition).

Phosphorylation of EphB2 Y504 and a negative charge at Y504 are required for interaction with the NMDAR^19^. To test how the charge of Y504 affects the EphB2–NMDAR interaction, PLA was performed on HEK293T cells transfected with EphB2 WT, EphB2 Y504E, or EphB2 Y504F, and WT Myc-GluN1, GluN2B, and EGFP (Fig. 6a). As expected, cells transfected with the more positively charged EphB2 phospho-null mutant (Y405F) had significantly fewer PLA puncta than both WT EphB2 and the more negatively charged phosphomimetic (Y504E) EphB2 (Fig. 6b, ****p < 0.0001, ANOVA). To specifically test the role of a negative charge at EphB2 Y504 in relation to the GluN1 charge mutants, PLA was performed on HEK293T cells transfected with EphB2 Y504E, GluN2B, EGFP and Myc-GluN1 WT or GluN1 point mutants (Fig. 6c, e). Consistent with the model that the negative charge of phosphomimetic EphB2 Y504E interacts with the positive potential of the GluN1 NTD hinge region, mutations in GluN1 that result in larger negative shifts of surface potential interact with EphB2 Y504E less well than those with smaller differences (Fig. 6d, f, WT vs. N273A, *p < 0.05; WT vs. R337D, ***p < 0.005; WT vs. N273A/R337D, ****p < 0.0001; ANOVA). Unfortunately, mutations of Y504 to a positively charged amino acid failed to reach the cell surface. Together these results suggest that the positively charged hinge region of GluN1 interacts with the negatively charged phosphorylated Y504 of EphB2 and this interaction is disrupted when GluN1 is mutated to become more negatively charged.

**Fig. 7:**
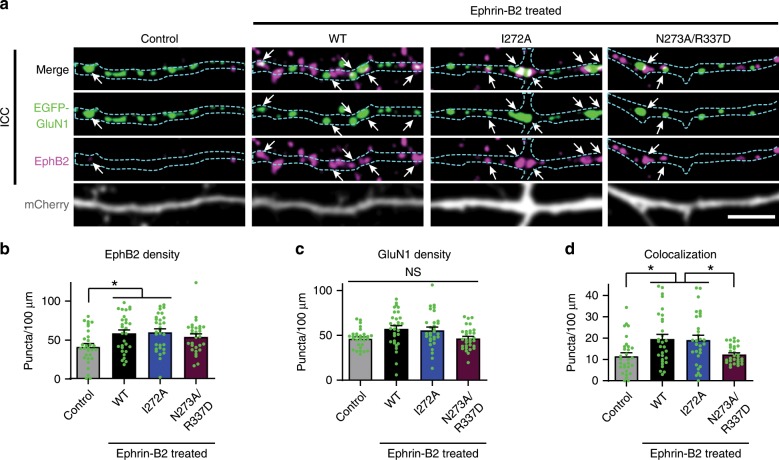
GluN1 charge mutants show disrupted EphB–NMDAR interaction in neurons. **a** Representative images of dendrites of DIV6-9 cortical neurons transfected with EGFP–GluN1 (WT, I272A, or N273A/R337D), mCherry, GluN2B, and CRISPR constructs to knock out endogenous GluN1. The control condition is WT GluN1-transfected treated with control reagents instead of ephrin-B2. The other three conditions were treated with ephrin-B2 for 45 min to induce the interaction between the NMDAR and EphBs. Top row panels show the colocalization between EGFP–GluN1 and EphB2 in white (arrows indicate examples of colocalized puncta). Second row panels show the high contrasted images of EGFP–GluN1 in green. Third row panels show endogenous EphB2 in magenta. Last row panels show mCherry. Outlines of morphology in cyan. Scale bar = 5 µm. **b** Quantification of EphB2 density (puncta number per 100 µm) in indicated groups (*p < 0.05, ANOVA; green dots represent *n* = 30 cells for each condition). **c** Quantification of EGFP–GluN1 density (puncta number per 100 µm) in indicated groups (*p* = 0.0128, ANOVA followed by Tukey’s; comparisons showed no significant differences; green dots represent *n* = 30 cells for each condition). **d** Quantification of the effects of the different GluN1 mutants on colocalization (puncta number per 100 µm) between GluN1 and EphB2 (*p < 0.05, ANOVA; green dots represent *n* = 30 cells for each condition).

Because the N273A/R337D GluN1 mutant appeared to have the most negative surface potential and had the strongest effect on the EphB2–NMDAR interaction (Fig. 5), we next asked whether it might disrupt the EphB2–NMDAR interaction in neurons. Surface staining showed that, following molecular replacement of GluN1, mutant (I272A and N273A/R337D) and WT GluN1 were localized to the neuron surface (Supplementary Fig. 9a, b). To test the effects of the mutants on the EphB2–NMDAR interaction, molecular replacement was conducted with WT EGFP–GluN1, I272A or N273A/R337D GluN1 mutants. Neurons were co-tranfected with GluN2B and mCherry. EphB clustering was induced with activated ephrin-B2^13^ and colocalization of endogenous EphB2 and EGFP–GluN1 was determined^19,45^. Ephrin-B2 activation resulted in clustering of EphB2 in all conditions (Fig. 7a, b). GluN1 and EphB2 puncta density were the same across the three ephrin-treated groups (Fig. 7b, c), suggesting that the effects on colocalization were not due to changes in EphB2 or NMDAR expression or puncta number. Ephrin treatment of WT GluN1 and GluN1 I272A-transfected neurons resulted in increased EphB2–GluN1 co-clustering compared to control (Fig. 7a, d, *p < 0.05, ANOVA). In contrast, the N273A/R337D GluN1 charge mutant was significantly less well colocalized with EphB2 compared to WT GluN1 (Fig. 7a, d, *p < 0.05, ANOVA). Together, these data suggest that the positive surface charge provided by N273 and R337 is necessary for the EphB–NMDAR interaction in neurons.

### Impact on synaptic stability of the NMDAR

**Fig. 8:**
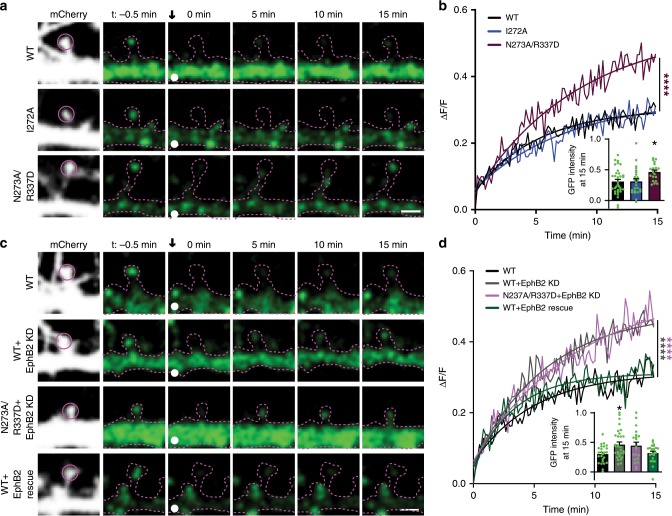
GluN1 charge mutants show increased mobility due to disrupted EphB–NMDAR interaction. **a** Representative FRAP images at different time points of DIV21–23 cortical neurons transfected with EGFP–GluN1 (WT, I272A, or N273A/R337D) together with GluN2B, CRISPR construct targeting endogenous GluN1, and mCherry. Recovery of bleached spine puncta (magenta circle) was monitored for 15 min at 10s intervals. Scale bar = 2 µm. **b** Quantification of the recovery curve of different GluN1 mutants in DIV21–23 cortical neurons. Graphs represent mean intensity and show fit (****p < 0.0001, Kolmogorov–Smirnov (KS) nonparametric test). Inset: Quantification of the mobile fraction of EGFP–GluN1 spine puncta at 15 min after photobleaching in EGFP–GluN1 mutant transfected cells compared to WT EGFP–GluN1 in DIV21–23 cortical neurons (*p < 0.05, ANOVA; green dots represent WT *n* = 33 puncta; I272A *n* = 12; N273A/R337D *n* = 12). Error bars show S.E.M. **c** Representative FRAP images at different time points of DIV21–23 cortical neurons transfected with EGFP–GluN1 (WT, WT+EphB2 knockdown, N273A/R337D + EphB2 knockdown, WT+EphB2 knockdown rescued by RNAi insensitive EphB2) together with GluN2B, CRISPR construct targeting endogenous GluN1, and mCherry. FRAP was conducted on serveral puncta in different dendritic branches. Recovery of bleached spine puncta (magenta circle) was monitored for 15 min at 10s intervals. Scale bar = 2 µm. **d** Quantification of the recovery curve of different GluN1 mutants in DIV21–23 cortical neurons. Graphs represent mean intensity and show fit (****p < 0.0001, Kolmogorov–Smirnov (KS) nonparametric test). Inset: Quantification of the mobile fraction of EGFP–GluN1 spine puncta at 15 min after bleaching in EGFP–GluN1 mutant transfected cells compared to WT EGFP–GluN1 in DIV21–23 cortical neurons (*p < 0.05, ANOVA; green dots represent WT *n* = 33 puncta; WT+EphB2 K.D. *n* = 12; N273A/R337D+EphB2 K.D. *n* = 21; rescue *n* = 22). Error bars show S.E.M.

Disrupting the EphB2–NMDAR interaction by mutating the hinge region of GluN1 results in increased mobility of NMDARs found in dendritic spines. We next asked whether the GluN1 N273A/R337D charge mutant is sufficient to increase the mobility of the NMDAR in dendritic spines. To test this, neurons were transfected with either WT EGFP–GluN1, EGFP–GluN1 I272A, or EGFP–GluN1 N273A/R337D, and GluN2B, mCherry, and GluN1 CRISPR. No significant differences were found between levels of WT or mutant EGFP–GluN1 in the dendritic shaft (Supplementary Fig. 9c, d), but there was a decrease in spine density in N237A/R337D expressing neurons (Supplementary Fig. 9e, f). EGFP–GluN1 mobility in dendritic spines was examined by FRAP, and images of EGFP–GluN1 puncta were collected once every 10 s for 15 min after bleaching. Fluorescence recovery of both WT and I272A EGFP–GluN1 was indistinguishable (Fig. 8a, b). In contrast, EGFP–GluN1 N273A/R337D recovered significantly more than WT (Fig. 8b, ****p < 0.0001, KS test; 15 min time point, *p < 0.05, ANOVA). These results suggest converting the surface charge in the hinge region of the GluN1 NTD increases NMDAR mobility at spines.

To test whether the increased mobility of EGFP–GluN1 N273A/R337D is caused by disruption of the EphB2–NMDAR interaction, we examined the impact of knocking down endogenous EphB2 in neurons transfected with WT or N273A/R337D EGFP–GluN1. If N273 and R337 are responsible for the EphB–NMDAR interaction and the stability of the NMDAR, then, following EphB2 knockdown, the WT but not the EGFP–GluN1 N273A/R337D mutant should show increased recovery after bleaching. To avoid potential confounds due to changes in synapse number, EphB2 was knocked down at DIV14, when knockdown of EphB2 has no effect on synapse density^46^. Consistent with this model, there was no effect on spine density along the dendrite when EphB2 was knocked down with a validated EphB2 shRNA construct^17,46^ at DIV14 in neurons expressing EGFP–GluN1 WT, GluN2B, mCherry, and GluN1 CRISPR (Supplementary Fig. 9e, f). We tested whether EphB2 knockdown would result in increased recovery of WT NMDARs in spine synapses using our molecular replacement strategy. Knockdown of endogenous EphB2 with a validated shRNA^17,46^ significantly increased WT EGFP–GluN1 mobility in dendritic spine synapses (Fig. 8c). This effect was rescued by expressing shRNA-insensitive EphB2 (Fig. 8c, d, ****p < 0.0001, KS test; 15 min time point, *p < 0.05, ANOVA). Consistent with previous studies showing that EphB2 is required for proper synaptic NMDAR localization^13,19^, these data indicate that NMDAR mobility is regulated by EphB2.

If the loss of the EphB–NMDAR interaction mediates the increase in mobility of the negatively charged GluN1 NTD mutants, we expect that knockdown of EphB2 should result in no further increase in EGFP–GluN1 N273A/R337D mobility in those neurons. Consistent with this model, knockdown of EphB2 in neurons expressing N273A/R337D EGFP–GluN1 resulted in no additional increase in recovery (Fig. 8c, d, ****p < 0.0001, KS test). Together, these data suggest that the positive charge of the hinge region of the GluN1 NTD is necessary for the EphB-NMDAR interaction and that this interaction regulates mobility of NMDARs.

### The EphB–NMDAR interaction is charge-dependent

**Fig. 9:**
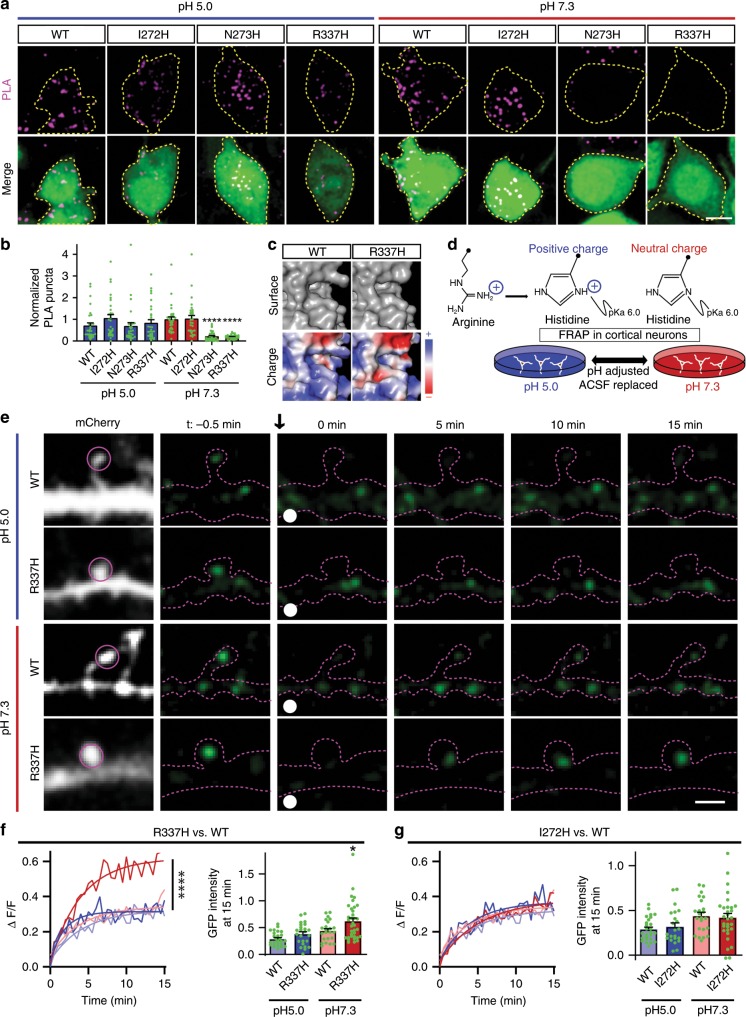
pH-sensitive histidine mutants in the hinge region elucidate a charge-dependent mechanism. **a** Representative images of PLA results in HEK293T cells. HEK293T cells were transfected with the indicated Myc-GluN1 single point mutants, together with GluN2B, FLAG-EphB2, and EGFP. Cells were treated with media at either pH 5.0 or pH 7.3 for 30 min before PLA. Upper panels: PLA signal alone. Lower panels: merge of EGFP in green and PLA signal in magenta. Scale bar = 10 µm. **b** Quantification of the effects of GluN1 mutants and pH on PLA puncta number (****p < 0.0005, ANOVA; green dots represent *n* = 30 cells for each condition). **c** Surface representation models (top) and charge maps (bottom) of WT GluN1 and R337H GluN1 predicted at neutral pH. **d** Model of experimental design. The same neurons were imaged for both pH conditions. **e** Representative FRAP images at different time points of DIV21–23 cortical neurons transfected with EGFP–GluN1 (WT or R337H) together with GluN2B, CRISPR construct targeting endogenous GluN1, and mCherry. FRAP of GluN1 puncta was conducted in the same neurons at pH 5.0 (H-positive) and pH 7.3 (H-neutral) and the order of pH presentation varied. Recovery of bleached spine puncta (magenta circle) was monitored for 15 min at 10s intervals. Scale bar = 2 µm. **f** Left: Quantification of the recovery curve of GluN1 WT or R337H mutant in ACSF at pH 7.3 and pH 5.0 in DIV21–23 cortical neurons. Graphs represent mean intensity and show fit (****p < 0.0001, Kolmogorov–Smirnov (KS) nonparametric test). Right: Quantification of the mobile fraction of EGFP–GluN1 spine puncta at 15 min after photobleaching (*p = 0.0398, WT-pH7.3 vs. R337H-pH7.3; ANOVA; green dots represent WT-pH5.0 *n* = 26 puncta; R337H-pH5.0 *n* = 24; WT-pH7.3 *n* = 23; R337H-pH7.3 *n* = 35). Error bars show S.E.M. **g** Left: Quantification of the recovery curve of GluN1 WT or I272H mutant in ACSF at pH7.3 and pH5.0 in DIV21–23 cortical neurons. Graphs represent mean intensity and show fit. (*p* = 0.8148, WT-pH7.3 vs. I272H-pH7.3, Kolmogorov–Smirnov (KS) nonparametric test). Right: Quantification of the mobile fraction of EGFP–GluN1 spine puncta at 15 min of FRAP (*p* = 0.9839; WT-pH7.3 vs. I272H-pH7.3; ANOVA; green dots represent WT-pH5.0 *n* = 26 puncta; I272H-pH5.0 *n* = 22; WT-pH7.3 *n* = 23; I272H-pH7.3 *n* = 30). Error bars show S.E.M.

The predicted structure of the charged hinge domain indicates that mutation of R337 results in modification of a feature formed by the arginine side chain (Figs. 5a and 9c). To achieve a dynamically modifiable surface charge, we took advantage of the p*K*_a_ of the side chain of histidine (Fig. 9d). The p*K*_a_ of histidine is 6.0. At a pH of 7.3, the deprotonated form of histidine is dominant (95% deprotonated) and the charge of histidine is neutral. At a pH of 5.0, the imidazole group of histidine is protonated (91% protonated) and is positively charged. Substituting histidine at GluN1 hinge region residues should generate a pH-sensitive molecular switch for the EphB-NMDAR interaction^48–50^. Because low pH (<6.0) would also alter the p*K*_a_ of any exposed histidine residues in the extracellular domain, and is known to result in rearrangements of the NMDAR ectodomain^51^, we generated three histidine point mutants in the GluN1 hinge region: I272H, N273H, and R337H. We expect that the WT and I272H mutant GluN1 should interact at both pH 5 and pH 7.3, controlling for the effects of rearrangements. If the EphB–NMDAR interaction is mediated by a positive surface charge in the hinge region, N273H and R337H mutants should interact with EphB2 at pH 5.0 but not at the physiological histidine-neutral pH 7.3.

To test whether the charge of the histidine mutants affect the interaction with EphB2, PLA was performed under two different pH conditions (pH 5.0 and pH 7.3). HEK293T cells were transfected with either WT Myc-GluN1 or each of the GluN1 histidine mutants (I272H, N273H, and R337H), together with GluN2B, EphB2, and EGFP (Fig. 9a, b). Transfected HEK293T cells were placed in either pH 5.0 (H-positive) or pH 7.3 (H-neutral) cell culture media for 20 min and then fixed. All GluN1 mutants expressed and were found at the surface at similar or higher levels compared to WT GluN1 in HEK293T cells at both pH 5.0 and pH 7.3 (Supplementary Fig. 10a–c). PLA was conducted for the extracellular domains of GluN1 (anti-Myc) and EphB2 (anti-EphB2) without permeabilization. As expected if positive charge is necessary for the interaction, there were no significant differences in PLA puncta density between conditions at pH 5.0 (H-positive) (Fig. 9a, b). Moreover, although there may be rearrangements of the extracellular domain at low pH that may disrupt NMDAR channel function^51^, there were no effects of pH on PLA puncta density in WT or I272H GluN1-transfected cells, suggesting that pH does not affect the ability of EphB2 and the NMDAR to interact (Fig. 9a, b. WT, *p* = 0.7464; I272H, *p* = 0.9999; ANOVA). However, in both N273H and R337H mutant GluN1 conditions at physiological pH (pH 7.3, H-neutral), the number of PLA puncta was significantly reduced compared to WT or I272H GluN1 (Fig. 9a, b. ***p < 0.0005, ANOVA). These data suggest that a positive surface potential in the hinge region at amino acid positions 273 and 337 of the GluN1 NTD is necessary for the EphB–NMDAR interaction.

Disrupting the EphB2–NMDAR interaction by mutating the hinge region of GluN1 results in increased mobility of NMDARs found in dendritic spines. We next asked if we could change the mobility of GluN1 by dynamically changing the charge of the hinge region in neurons. Primary cortical neurons were transfected with either WT, 1272H, or R337H EGFP–GluN1, and GluN2B, mCherry, and GluN1 CRISPR. Calcium imaging in live neurons revealed that the histidine point mutation does not affect channel function at pH 7.3 (Supplementary Fig. 5). Surface staining showed that both mutant and WT GluN1 receptors were localized to the neuron surface at both pH 5.0 and pH 7.3 (Supplementary Fig. 10d, e). In addition, the acidity of ACSF at pH 5.0 quenches the EGFP signal, resulting in less average fluorescence intensity compared to pH 7.3. These data suggest the EGFP tag is exposed to the extracellular environment and GluN1 is surface localized (Supplementary Fig. 10d, e and f–i, *p < 0.05, paired *t*-test)^52^.

To dynamically determine the impact of charge of the hinge region on the mobility of GluN1 in individual cortical neurons, FRAP was performed on EGFP–GluN1 puncta in dendritic spines of neurons placed into pH 5.0 or 7.3 ACSF immediately prior to imaging. Images were acquired every 10 s for 15 min to examine EGFP–GluN1 recovery. The pH of the ACSF was then changed and new puncta in the same neuron were selected for FRAP. Results were the same regardless of the order of pH presentation.

At pH 5.0 (H-positive), recovery was indistinguishable between WT EGFP–GluN1 and all of the histidine mutants (Fig. 9e–g). These data suggest that under these conditions, NMDAR channel function is not required for receptor stabilization at synaptic sites. Consistent with the importance of charge for the EphB–NMDAR interaction, at physiological pH (pH 7.3, H-neutral) when histidine loses its positive charge, EGFP–GluN1 R337H recovered significantly more than both I272H and WT EGFP–GluN1 (Fig. 9e–g, ****p < 0.0001, KS test; 15 min time point, *p < 0.05, ANOVA). There were no differences between WT and I272H EGFP–GluN1 at either pH (Fig. 9e–g, dark red and dark blue curves). These data suggest that the charge of R337, not the side chain of the arginine is critical for NMDAR mobility. Thus, the EphB–NMDAR interaction is mediated by a charge-dependent mechanism and is likely an important regulator of the dynamic stability of the NMDAR at spines.

## Discussion

The mechanisms that regulate the synaptic localization of the NMDAR have been the subject of intense study. Here, we show that the EphB–NMDAR interaction is involved in regulating NMDAR localization to dendritic spine synapses and define the structural interface (N273 and R337) of the GluN1 NTD required for the EphB–NMDAR interaction. EphBs are extracellularly phosphorylated at Y504 upon activation by ephrin-B, which is required for the EphB–NMDAR interaction^19^. Disruption of the interaction by changing the surface charge of this region increases NMDAR mobility in dendritic spines. These findings indicate that, similarly to other protein–protein intracellular interactions that are regulated by phosphorylation, the extracellular EphB2-binding domain on GluN1 likely consists of a positively charged domain to coordinate with the negative charge of a p*Tyr in the EphB2 FN3 domain. Given that a number of other synaptic proteins contain extracellular p*Tyr residues in homologous FN3 domains^19^, these findings suggest a conserved general charge-based mechanism for interaction of the extracellular domains of synaptic proteins.

Phosphorylation of Y504 in the extracellular domain of EphB2 is necessary and sufficient to induce the EphB–NMDAR interaction^19^. Extracellular kinases that mediate phosphorylation of extracellular tyrosines have recently been identified^26^. However, how phosphorylation of these sites might mediate interactions between proteins has not been elucidated. Our data suggest that similar to intracellular interactions, positively charged domains appear to be important for recognition and p*Tyr binding. Interestingly, the positively charged EphB-binding domain on GluN1 requires an arginine residue perhaps to coordinate the negative charge of p*Tyr at Y504. Similarly, both SH2 and PTB intercellular p*Tyr-binding domains contain arginine residues^23,24^. However, the structure of the GluN1 hinge region differs from the defined SH2 and PTB domains, with the GluN1 hinge region containing flexible linker structures rather than structurally rigid β-sheets. This suggests the GluN1 hinge region may define a p*Tyr interacting domain.

All ionotropic glutamate receptors contain an NTD with a similar structure that appears to be a binding hub for extracellular protein–protein interactions and allosteric modulators^10^. Indeed, ionotropic glutamate AMPA receptors (AMPAR) lacking the extracellular NTD in the GluA2 subunit exhibit increased mobility in synapses^53^. The NTD of other glutamate receptors seems to mediate extracellular interactions with a number of proteins, such as N-cadherin^54^ and neuronal pentraxins (NARP and NP1) with AMPA receptor subunits^55–57^ and Cbln1 with GluRδ2^58,59^. Our study is an example of an endogenous protein interaction with the hinge region of the GluN1 NTD. Additional effort will be needed to determine whether charge-based mechanisms are responsible for other glutamate receptor interactions.

The extracellular subunits that comprise the NMDAR share similar structures, including an LBD located near the cell membrane and an NTD. The NTDs of NMDAR subunits can modulate NMDAR channel function via interaction with allosteric regulators^7^. The binding partners of the GluN2 subunits are much better characterized than those of the GluN1 subunit. The NTD of the GluN2B subunit of the NMDAR has at least three distinct binding sites for allosteric regulators, with zinc and ifenprodil functioning as allosteric inhibitors and polyamines acting to enhance NMDAR receptor activity^9^. Interestingly, spermine, a positive allosteric modulator of GluN2B-containing NMDARs, binds to the hinge region and lower lobe of the GluN1–GluN2B NTD interface in a charge-dependent manner^8,9^.

NMDARs move into and out of synaptic sites^16^. Single particle tracking and FRAP show that there are barriers to the lateral diffusion of NMDARs that help to maintain the position of the receptors within the postsynaptic density (PSD)^11,60–62^. Our data support a model in which the EphB–NMDAR interaction regulates NMDAR mobility and helps to anchor NMDARs at synaptic sites. Anchoring of the NMDAR appears to be important for maintaining spine density. Blocking the EphB–NMDAR interaction results in a reduction in spine density that is dependent on expression of EphB2, suggesting that EphB2 may act as a punishment signal in the absence of an interaction with GluN1, driving a loss of spines. Regardless, EphB2, which is found in the core of the PSD^63^, provides a potential hub for protein–protein interactions to selectively maintain the NMDAR in the core of the PSD and, through the transsynaptic EphB2–ephrin-B interaction, localized adjacent to presynaptic release sites. This mechanism of NMDAR stabilization may reveal new avenues of research towards understanding pathological conditions involving dysfunction of the NMDARs at the synapse and extracellular protein–protein interactions^19,20^.

## Methods

### Animals

All animal procedures were approved by the Institutional Animal Care and Use Committee of Thomas Jefferson University (01286 and 01289). Long Evans rats (Charles River) were used for preparing dissociated cortical neuron cultures as described below^17,46^. CD-1 mice (Charles River) were used for preparing synaptosomes.

### Synaptosomes

Synaptosomes were prepared from postnatal day 21 (P21) wild-type CD-1 mice^31^. Brains were homogenized on ice in 0.32 M sucrose, 4 mM HEPES, pH 7.4, containing a protease inhibitor cocktail (Sigma) and 1 mM PMSF. After removing the nuclear fraction by centrifugation at 1000×*g* for 15 min at 4 °C, non-synaptic fractions were further centrifuged at 10,000×*g* at 4 °C to obtain the crude synaptosomal fraction. Purified synaptosomes were obtained by centrifugation of this fraction through a gradient of 1.2 M sucrose to 0.8 M sucrose. To plate frozen synaptosomes,^64^ coverslips were prepared as follows. Using a PEI Stock Solution (1:15 Stock: 3.3 mL 50% (wt/vol) PEI in 46.7 mL dH_2_O), 50 mL 1:15,000 PEI dilution was prepared (50 μL stock in 50 mL dH_2_O). 400 μL PEI dilution was added to glass coverslips in a 24-well plate to incubate at 37 °C overnight (or up to 48 h). PEI was removed from coverslips and coverslips were  allowed to dry at 37 °C for 30 min. Dried coverslips were kept at 4 °C while preparing synaptosomes. Frozen synaptosomes were thawed on ice and diluted to 20 ng/mL in SET buffer (0.32 M Sucrose, 1 mM EDTA, 5 mM Tris, pH 7.4) with 250 μM DTT. Diluted synaptosomes were pipetted onto prepared cover slips at a concentration of 8 μg per cover slip. Cover slips were centrifugated in a 24-well plate at 1500×*g* for 30 min at 4 °C and then immunostained.

### Expression constructs

EGFP–GluN1 was  purchased from Addgene (Watertown, MA; ID 45446). For neuronal transfection, the EGFP–GluN1 fragment was cloned into a synapsin promoter-containing pLV-hSyn vector to ensure its expression only in neurons. Single point mutations were introduced using sequence-specific primers (see Primers in Supplement) and site-directed mutagenesis. For HEK293T PLA experiments, the GluN1 EGFP tag was switched to a Myc tag using sequence-specific primers. FLAG-tagged EphB2 and HA-tagged GluN2B were generated and used previously^13^.

### HEK293T cell culture and transfection

HEK293T cells (Greenberg lab, originally from ATCC) were maintained in DMEM (Invitrogen), 10% fetal bovine serum (Atlanta Biologicals), 1% penicillin–streptomycin (Invitrogen), and 1% glutamine (Invitrogen). Cells were transfected with Lipofectamine 2000 (Invitrogen) according to the manufacturer’s protocol or with calcium phosphate^65^. Briefly, the pH of 2X HeBS (274 mM NaCl, 10 mM KCl, 1.4 mM Na_2_HPO_4_·7H_2_O, 15 mM d-glucose, 42 mM HEPES) was adjusted by NaOH to yield pHs ranging from 7.03, 7.05, 7.07, 7.09, 7.11, to 7.14. 2X HeBS was filter-sterilized, aliquoted and stored at 4 °C. Each pH was tested to determine which provided the best transfection efficiency. To prepare transfection mixture, 70 µL of HEPES-buffered dH_2_O (2.5 mM HEPES) was added to an Eppendorf tube, 8.65 µL of 2.5 M CaCl_2_ was added to the bottom of the HEPES-containing tube, then plasmid DNA was added on top of the CaCl_2_–HEPES mixture and mixed by adding 86.5 µL of the most pH effective 2X HeBS. Precipitation was initiated by bubbling the transfection mixture with a pipette 8–10 times. The mixture was immediately added to HEK cells (one transfection mixture per one well of a six-well plate) dropwise. Transfected cells were immunostained, imaged or lysed 16–24 h after transfection. Myc-GluN1, GluN2B, FLAG-EphB2, and EGFP constructs were co-transfected for HEK293T PLA experiments. 50 µM APV (Tocris Bioscience) and 10 µM MK801 (Tocris Bioscience) were added after transfection to prevent excitotoxicity.

### Primary neuronal culture

Rat cortical cultures were prepared from embryonic day 17 (E17) rat embryos^17,46^. Embryos were harvested and brains were isolated in ice-cold 20 mM HEPES-buffered Hank’s balanced salt solution (HBSS). Meninges were removed using fine forceps. The striatum and hippocampi were separated and discarded and cortices were collected. Cortices were incubated with 10 µg/mL papain (Worthington Biochemical Corporation) in HBSS for 4 min at 37 °C. After three washes in HBSS with 0.01 g/mL trypsin inhibitor (Sigma), the cortices were gently triturated with a fire-polished glass Pasteur pipette 5–10 times to obtain a homogeneous cell suspension. Bubbling was avoided and the pipette was maintained within the cell suspension during the trituration. Neurons were plated on poly-d-lysine (BD Biosciences, Bedford, MA) and laminin (BD Biosciences)-coated glass coverslips (12 mm; Bellco Glass, Vineland, NJ) in 24-well plates (Corning Life Sciences, Lowell, MA). For FRAP experiments, neurons were plated on glass bottom dishes (35 mm, GBD00002-200, Cell E&G). Standard density for neuronal cultures was 6 × 10^5^ cells/cm^2^. Neurons were cultured in Neurobasal media (Invitrogen, Carlsbad, CA) supplemented with B-27 (Invitrogen), 1% glutamine (Invitrogen), and 1% penicillin–streptomycin (Invitrogen). Neurons were transfected using Lipofectamine 2000 (Invitrogen, see below)^17,46^. For neurons transfected with NMDARs, 100 µM APV was added every 2 days after transfection.

### Neuronal transfection

Neurons were transfected either at day in vitro 0 (DIV0) in suspension^17,46^ or DIV3 using Lipofectamine 2000 (Invitrogen, Carlsbad, CA) and processed for ICC or transfected at DIV14 using Lipofectamine 2000 (Invitrogen) and processed for FRAP at DIV21-23. Transfection mixture was prepared (per coverslip) as follows: 0.5 µL Lipofectamine 2000 was added to 50 µL neurobasal medium (without supplement) in a polystyrene tube (USA Scientific). DNA was added to 50 µL neurobasal medium (without supplement) in an Eppendorf tube. After 5 min, the DNA mixture was slowly added to Lipofectamine mixture dropwise. The combined mixture was mixed by bubbling two times and incubated at room temperature for 15 min. While the transfection mixture incubated, the conditioned media was removed from the neuronal cultures, saved at 37 °C, and replaced with 300 µL of warm neurobasal medium (without supplement) per one well of a 24-well plate and kept at 37 °C until ready for transfection. The transfection mixture was added dropwise to the neuronal culture and incubated for 2 h at 37 °C. Then transfection media was replaced with filter-sterilized warm conditioned media and the neuronal culture was returned to the incubator. For RCaMP experiments, Synapsin–EGFP–GluN1 was co-transfected with RCaMP (pAAV.Syn.NES-jRCaMP1b.WPRE.SV40)^44^, pFUG–HA–GluN2B, and GluN1 CRISPR constructs. For all other experiments, Synapsin–EGFP–GluN1 was co-transfected with pFUG–HA–GluN2B, CAG–mCherry, and GluN1 CRISPR constructs. GluN1 CRISPR (pX330-U6-Chimeric_BB-CBh-hSpCas9) was obtained from Addgene and transfected as described above and is a published construct^42,43^. GluN2B was chosen to be co-expressed with GluN1 because GluN2B is expressed in young neurons, the synaptic incorporation of GluN2B-containing receptors is not limited by synaptic transmission nor enhanced by increased subunit expression, and GluN2 subunit expression promotes NMDAR surface localization of exogenously expressed receptors^66^.

### Proximity ligation assay

PLA experiments were performed using the Duolink In Situ Orange Detection Kit (DUO 92105, Sigma) based on manufacturer’s protocol. For pH-specific experiments, HEK293T cells were treated with media at either pH 5.0 or pH 7.3 for 30 min before fixation. PLA images were collected using a Leica TCS SP8 confocal with a ×63 objective lens. Pictures shown were Z stacks of whole cells.

### Immunoprecipitation

Immunoprecipitations were performed on ice^13,19^. Transfected HEK293T cells were lysed in 350 µL (per well for six-well plates) RIPA buffer (20 mM Tris–Cl pH 7.5, 140 mM NaCl, 2 mM EDTA, 10 mM NaF (Sigma), 1% NP40 (Thermo Scientific), 0.5% sodium deoxycholate, 1 mM Na_3_VO_4_, 1 mM PMSF, and protease inhibitor cocktail (all from Sigma)). Cell lysates were harvested and centrifuged at 16,000×*g* for 25 min at 4 °C to pellet cellular debris. A fraction of the resulting supernatant (50 µL per well for 6-well plates) was removed as an input control. The remaining supernatant was incubated with rabbit polyclonal anti-FLAG to conjugate on ice for 1.5 h. Antibody-bound proteins were then isolated using protein-G agarose beads (Invitrogen) pre-blocked with BSA on a rotator at 4 °C for 1 h. Samples were then centrifuged and beads were washed three times in RIPA lysis buffer and two times in TBS-V. Immunoprecipitants were eluted from the agarose beads by adding boiling SDS-sample buffer containing 32% 2-Mercaptoethanol (Sigma) and 9% 1,4-Dithiothreitol (DTT, Sigma) and boiled at 70 °C for 10 min.

### Western blotting

Lysates from HEK293T cells were separated by SDS–polyacrylamide gel electrophoresis (SDS–PAGE) using 8% Tris–glycine gels and transferred onto 0.45 µm PVDF membranes (Millipore). Immunoblots were then blocked in 5% nonfat dry milk in TBS-T (150 mM NaCl, 10 mM Tris pH 8.0, 0.05% Tween-20). Blots were incubated with primary antibodies in blocking solution for 2 h at room temperature or overnight at 4 °C. HRP-conjugated secondary antibodies were used at 1:10,000 in blocking solution for 1 h at room temperature then visualized using ECL (PerkinElmer, Waltham, MA) and autoradiography film (HyBlot Film, Denville Scientific).

### Immunocytochemistry

For GluN1 surface staining, live neurons were stained for 10 min at 37 °C in artificial cerebrospinal fluid (ACSF, 140 mM NaCl, 5 mM KCl, 1 mM MgCl_2_, 2 mM CaCl_2_, 20 mM glucose, and 10 mM HEPES, pH 7.3)^67^ with anti-EGFP, then washed with ACSF once and fixed with 4% paraformaldehyde/2% sucrose in PBS. For live-cell surface staining in the pH-specific experiments, neurons were treated with media at either pH 5.0 or pH 7.3 for 30 min and then live cell-stained for 10 min with anti-EGFP in their respective pH-adjusted medias at 37 °C . After several washes, cells were fixed with 4% paraformaldehyde/2% sucrose and blocked with PBS containing 1% ovalbumin (Sigma) and 0.2% cold water fish skin gelatin (Sigma). After blocking, cells were incubated with secondary antibodies in the previously described blocking reagent for 45–60 min at room temperature. Cells were then washed three times in PBS before mounting on glass microscope slides (Fisher) with Aqua-Mount (Lerner, Kalamazoo, MI). For detection of EphB2 and EGFP–GluN1 via confocal microscopy, neurons were fixed with 4% paraformaldehyde/2% sucrose at room temperature. After several washes, cells were blocked with PBS containing 1% ovalbumin and 0.2% cold water fish skin gelatin (Sigma) for 45 min at room temperature, then incubated with primary antibody for 2 h at room temperature in the previously described blocking reagent. For staining of Myc-GluN1 and FLAG-EphB2 in HEK293T cells, cells were fixed in the same way as neurons. For pH-specific experiments,  HEK293T cells were treated with media at either pH 5.0 or pH 7.3 for 30 min before fixation. Following fixation with 4% paraformaldehyde/2% sucrose in PBS, cells were blocked and permeablized with PBS containing 1% ovalbumin, 0.2% cold water fish skin gelatin, and 0.01% saponin for 45 min at room temperature, then immunostained with primary antibodies in blocking reagent for 1 h at room temperature. After washing three times with PBS, cells were incubated with secondary antibodies in blocking reagent for 45–60 min at room temperature. Cells were then washed three times in PBS before mounting on glass microscope slides (Fisher) using Aqua-Mount (Lerner, Kalamazoo, MI).

### Calcium imaging

Experiments were conducted with a Leica TCS SP8 confocal scanning microscope. Cultured neurons on coverslips were removed from the culture dish, placed in an imaging perfusion chamber with low calcium (1 mM), no magnesium ACSF and imaged using a ×63 oil immersion lens (Leica). Cells co-transfected with EGFP–GluN1 and RCaMP (pAAV.Syn.NES-jRCaMP1b.WPRE.SV40)^44^ were imaged for 600 frames (2 min) at 5 frames per second. Low calcium, no magnesium ACSF containing 25 µM CNQX (Tocris, IL), 40 µM Nifedipine (Tocris, IL), and 1 µM Tetrodotoxin (Cayman, MI; ACSF*) was perfused onto the cells for 100 frames (20 s). After 100 frames, ASCF* was switched off and ASCF* plus 10 µM glutamate and 50 µM glycine was switched on for 50 frames (10 s). Glutamate was turned off and ACSF* was then switched back on for the remaining 450 frames (90 s). Each cell was imaged three times and response was averaged. ACSF* containing 100 µM APV (Tocris, IL) was then perfused on and the imaging process described above was repeated in the presence of APV to ensure cell response was NMDAR-specific.

### Fluorescence recovery after photobleaching (FRAP)

Experiments were conducted with a Leica TCS SP5 or SP8 confocal scanning microscope. Cultured neurons on coverslips were removed from the culture dish, placed in an imaging chamber with ACSF and imaged using a ×63 oil immersion lens (Leica). Photobleaching of GluN1–EGFP puncta in mushroom-shaped spines (or on dendritic shaft as indicated) was performed by the Leica bleach points algorithm associated with a Leica TCS SP5/SP8 confocal microscope using 100 ms of a 488 nm laser set at 20% power. For pH-specific FRAP experiments, neurons were grown in round glass bottom dishes (Cell E&G). pH-specific ACSF was removed and replaced with a pipette while the dish of neurons remained stationary between conditions. Imaging began within 5 min or less of media replacement. Cells were randomly imaged with either pH 7.3 first or pH 5.0 first. A different GluN1 puncta was imaged on the same cell after pH switch for paired comparisons. The first image of each of these experiments (before bleaching) was used to measure fluorescence intensity for pH quenching results.

### FRAP analysis

EGFP intensity of bleached puncta and three randomly selected unbleached puncta were quantified for each time point by selecting the regions of interest (ROIs) around selected puncta in every image in the time series^45^. The unbleached puncta served as a control for the photobleaching during acquisition. The recovery of bleached EGFP puncta was normalized to the average intensity of these control puncta at each time point. The recovery of bleached puncta (represented as EGFP intensity) was then determined using the equation: ((*F*_*t*_−*F*_0_)/(*F*_pre_−*F*_0_)), where *F*_*t*_ is the EGFP fluorescence measured at different time intervals, *F*_0_ is the EGFP fluorescence immediately after photobleaching at *t* = 0 s, and *F*_pre_ is the average EGFP fluorescence intensity before photobleaching. These values were then divided by (CtrlF_t_/CtrlF_pre_), where CtrlF_*t*_ is the EGFP fluorescence of control unbleached puncta measured at different time intervals and CtrlF_pre_ is the average EGFP fluorescence intensity of the first five frames of control unbleached puncta, to control for slight variations in focus.

### Antibodies

The following primary antibodies and dilutions were used: mouse monoclonal (IgG1) anti-GluN1 (1:500 (WB), BioLegend, clone R1JHL, cat# 828201, lot# B212895), goat polyclonal anti-EphB2 (1:1200 (ICC, PLA), R&D Systems, cat# AF467, lot# CVT0315041), mouse monoclonal (IgG1) anti-EphB2 (1:500 (WB), Invitrogen, clone 1A6C9, cat# 37-1700, lot# RD215698), rabbit polyclonal anti-Myc (1:3000 (ICC, PLA), Abcam, Cambridge, MA, cat# ab9103, lot# 2932489), mouse monoclonal (IgG2b) anti-GluN2B (1:500 (WB), Neuromab, UC Davis, Davis, CA, clone N59/36, cat# 75-101, lot# 455-10JD-82), mouse monoclonal (IgG2A) anti-PSD-95 (1:2500 (WB), Neuromab, UC Davis, Davis, CA, clone 28/43, cat# 75-028, lot# 455.7JD.22f), mouse monoclonal (IgG1) anti-Synaptophysin-1 (1:5000 (WB), Synaptic Systems, Gottingen, Germany, clone 7.2, cat# 101 111, lot# 101011/1-43), guinea pig polyclonal anti-vesicular glutamate transporter 1 (vGlut1; 1:5000 (WB, ICC), EMD Millipore, Temecula, CA, cat# AB5905, lot# 2932489), rabbit polyclonal anti-GFP (1:3000 (ICC), Life Technologies, cat# A6455, lot# 1736965), mouse monoclonal (IgG1) anti-GAPDH (1:500 (WB), EMD Millipore, Temecula, CA, cat# MAB374, lot# 2910381), rabbit polyclonal anti-Tubulin (1:10,000 (WB), Abcam, Cambridge, MA, cat# ab18251, lot# GR235480-2), rabbit polyclonal anti-actin (1:2000 (ICC), Sigma, cat# A2103, lot# 115M4865V), rabbit polyclonal anti-FLAG (1:1000 (IP, WB), Sigma, Cat# F7425, lot# 097M4882V). The following secondary antibodies were used: Donkey anti-mouse-HRP (1:10,000 (WB), Jackson ImmunoResearch, cat# 715-035-151, lot# 128396), Donkey anti-rabbit-HRP (1:10,000 (WB), Jackson ImmunoResearch, cat# 711-035-152, lot# 132960), Donkey anti-goat-HRP (1:10,000 (WB), Jackson ImmunoResearch, cat# 705-035-147, lot# 112876), Donkey anti-rabbit unconjugated (1:100 (ICC), Jackson ImmunoResearch, cat# 711-005-152 lot# 125861), Donkey anti-mouse AlexaFluor-488 (1:500 (ICC), Jackson ImmunoResearch, cat# 715-545-150, lot# 11603), Donkey anti-rabbit AlexaFluor-488 (1:500 (ICC), Jackson ImmunoResearch, cat# 711-545-152, lot# 126601), Donkey anti-goat Cy3 (1:500 (ICC), Jackson ImmunoResearch, cat# 705-166-147, lot# 107019), Donkey anti-rabbit Cy3 (1:500 (ICC), Jackson ImmunoResearch, cat# 711-165-152, lot# 123091), Donkey anti-guinea pig AlexaFluor-647 (1:500 (ICC), Jackson ImmunoResearch, cat# 706-605-148, lot# 116734), Donkey anti-goat AlexaFluor-647 (1:500 (ICC), Jackson ImmunoResearch, cat# 706-606-147, lot# 124186).

### Statistical analysis

Data are expressed as means with individual values overlaid as dots. Error bars represent standard error of the mean (S.E.M.). Statistical significance of differences among groups was determined by one-way analysis of variance (ANOVA) followed by Tukey’s post-hoc test or *t*-tests as noted in the figure legends. FRAP curves were compared using KS nonparametric test. Distribution of data was assumed to be normal, but this was not formally tested. Probability values of <5% were considered statistically significant with * representing *p* < 0.05, ** representing *p* < 0.01, *** representing *p* < 0.005, and **** representing *p* < 0.001 unless otherwise indicated. Data were collected from a minimum of three independent experiments.

### Reporting summary

Further information on research design is available in the Nature Research Reporting Summary linked to this article.

## Supplementary information

Supplementary Information

Reporting Summary

## Data Availability

Data supporting the findings of this manuscript are available from the corresponding author upon reasonable request. A reporting summary for this article is available as a Supplementary Information file. The source data underlying Figs. 1b, d, 2h, 3b–d, 4b, 5c, f, 6b, d, f, 7b, c, 8b, d, 9b, f, g and Supplementary Figs. 1e, 2d, f–g, 3c, 4b, d, 5b–f, 6c, d, g, 7c, d, g, h, 9b, d, f, 10b, c, e, g–i are provided as a Source Data file.

## Source data

Source Data
