# Supplementary material for: Positive surface charge of GluN1 N-terminus mediates the direct interaction with EphB2 and NMDAR mobility
Source: Nat Commun. 2020 Jan 29;11:570. doi: 10.1038/s41467-020-14345-6 (PMC6989673; doi:10.1038/s41467-020-14345-6)
Supplement: Supplementary file 1 — Supplementary Information [file 41467_2020_14345_MOESM1_ESM.pdf]

Positive surface charge of GluN1 N-terminus mediates the direct interaction with EphB2 and NMDAR mobility

Halley R. Washburn, Nan L. Xia, Wei Zhou, Yu-Ting Mao, Matthew B. Dalva

Supplementary Figures 1-10

Supplementary Figure 1: GluN1 and EphB2 interact in synaptosomes.

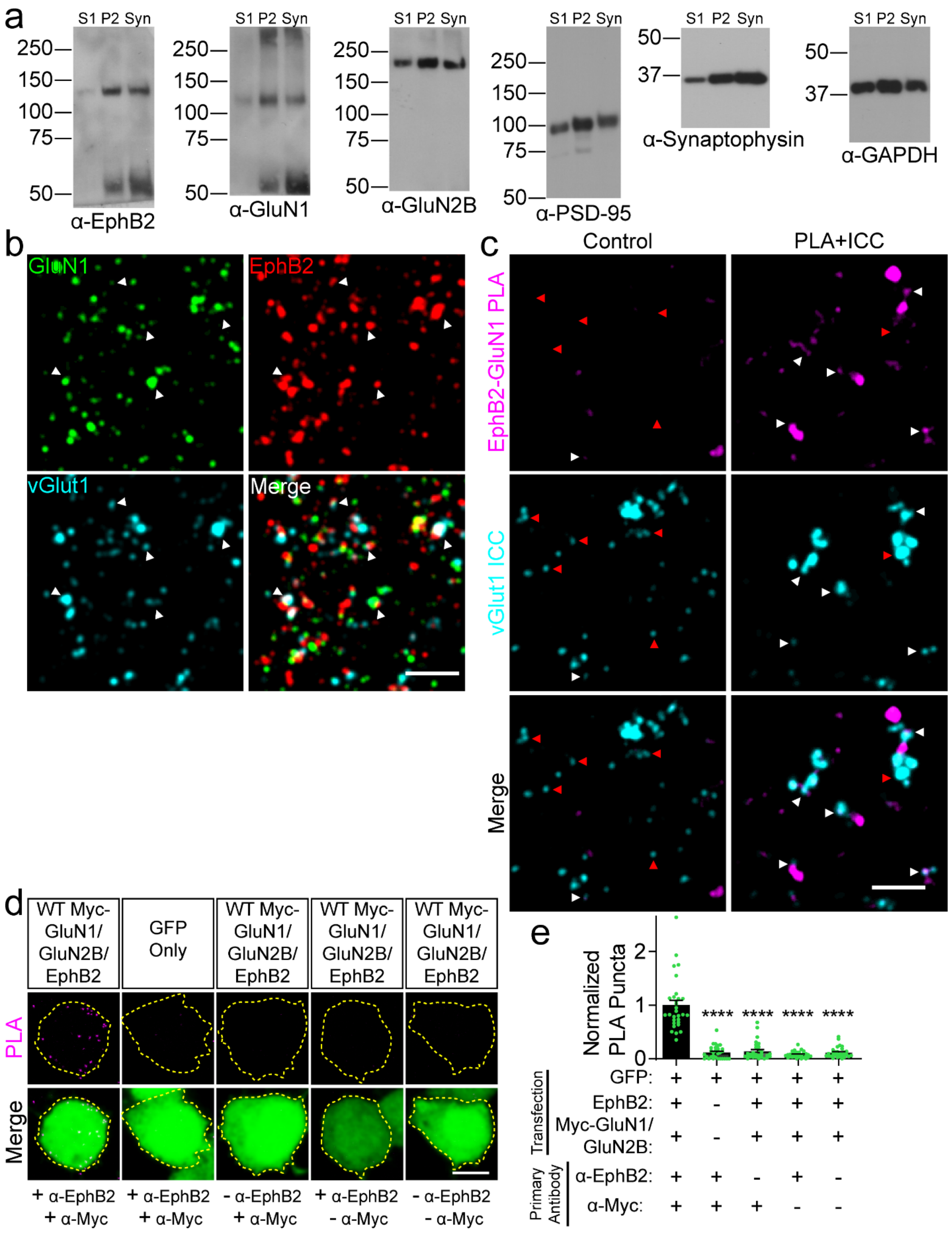

Supplementary Figure 1: a Western blots of lysates from synaptosomes prepared from WT CD1 mouse brain. Gels were loaded with non-synaptic (S1), crude synaptosomal (P2), and synaptosomal (Syn) fractions. Blots were probed with (from left to right): α-EphB2, α-GluN1, α-GluN2B, α-PSD-95 as a postsynaptic marker, α-Synaptophysin1 as a presynaptic marker, and α-GAPDH as a loading control. Complete blots are shown.

b Representative images of synaptosomes immunostained for GluN1 in green, EphB2 in red, and vGlut1 in cyan. Lower right panel shows merged image. White arrow heads indicate examples of triple colocalization. Scale bar = 5μm.

c Representative images of synaptosomes immunostained for vGlut1 in cyan (middle panels). PLA between EphB2 and GluN1 is shown in magenta (left panels). Right panel shows merged image. White arrow heads indicate examples of colocalization. Red arrow heads indicate examples of vGlut1 puncta without PLA puncta colocalized. Scale bar = 5μm.

d Representative images of the results of PLA assay controls in HEK293T cells. HEK293T cells were transfected with either WT Myc-GluN1, GluN2B, FLAG-tagged-EphB2, and EGFP or EGFP alone. Primary antibodies for either EphB2 or Myc-GluN1 were omitted as indicated below the images. The upper panels show PLA signal alone. The lower panels are the merged pictures with EGFP in green and PLA signal in magenta. Scale bar = 10μm.

e Quantification of PLA puncta number. PLA puncta number are quantified by counting the number of puncta per 100 μm^2 in EGFP$^+$ cells and normalizing to the average of the

WT condition (****$p<0.0001$, ANOVA followed by Tukey's; green dots represent n = 30 cells for each condition).

Supplementary Figure 2: Expression of GluN1 hinge mutants in HEK293T cells.

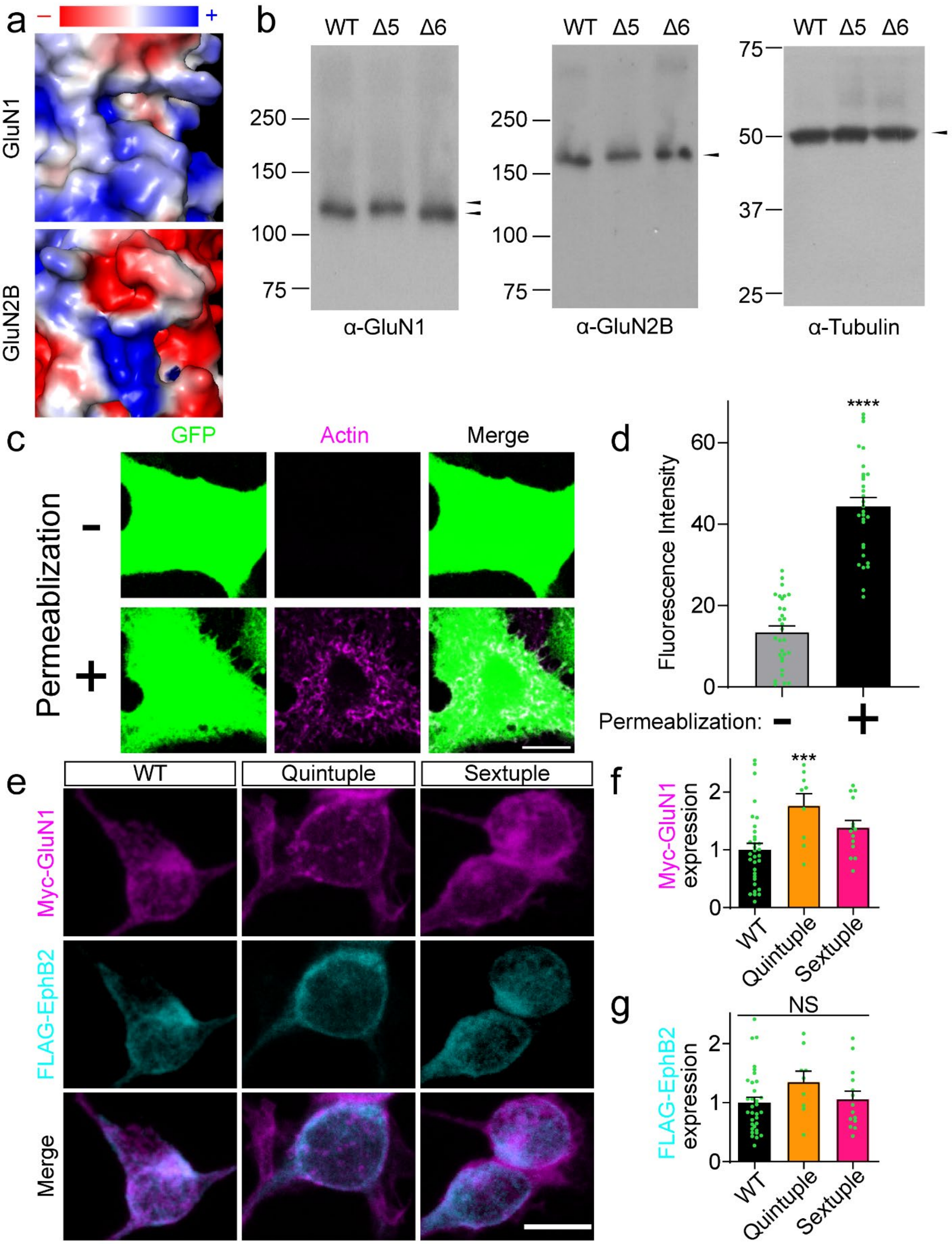

Supplementary Figure 2: a Charge map of the GluN1 (PDB: 4PE5) NTD hinge region (top) and GluN2B (PDB: 4PE5) NTD hinge region (bottom). The charge maps were generated using the Adaptive Poisson-Boltzmann Solver (APBS) plugin in PyMOL.

b Western blot of lysates from HEK293T transfected with GluN1 WT or mutants as indicated, and GluN2B. Lysate blots were probed with α-GluN1 (left), α-GluN2B (middle), and α-tubulin as a loading control (right). Arrows indicate appropriate bands. N350 mutation (Sextuple mutant) shows a lower apparent molecular weight due to predicted loss of glycosylation compared to GluN1 WT. Complete blots are shown.

c Representative images showing immunocytochemistry of actin with and without permeabilization as a control for surface staining. Actin is in magenta and EGFP is in green. Scale bar = 10μm.

d Quantification of actin fluorescence intensity. (****$p<0.0001$, unpaired t-test; green dots represent n = 30 cells for each condition).

e Representative images showing immunocytochemistry of Myc-GluN1 and FLAG-EphB2 expression. Myc-GluN1 mutants are in magenta and FLAG-EphB2 is in cyan. Scale bar = 10μm.

f Quantification of Myc-GluN1 expression level using fluorescence intensity. Values are normalized to the average of WT transfected group. Mutants are not less than WT (***$p=0.0036$, ANOVA followed by Tukey's; green dots represent WT n = 33 cells; Quintuple n = 9; Sextuple n = 14).

g Quantification of FLAG-EphB2 expression level using fluorescence intensity. Values are normalized to the average of WT transfected group. ($p=0.2198$, ANOVA followed by Tukey's; green dots represent WT n = 33 cells; Quintuple n = 9; Sextuple n = 14).

Supplementary Figure 3: Knocking down of endogenous GluN1 using CRISPR constructs.

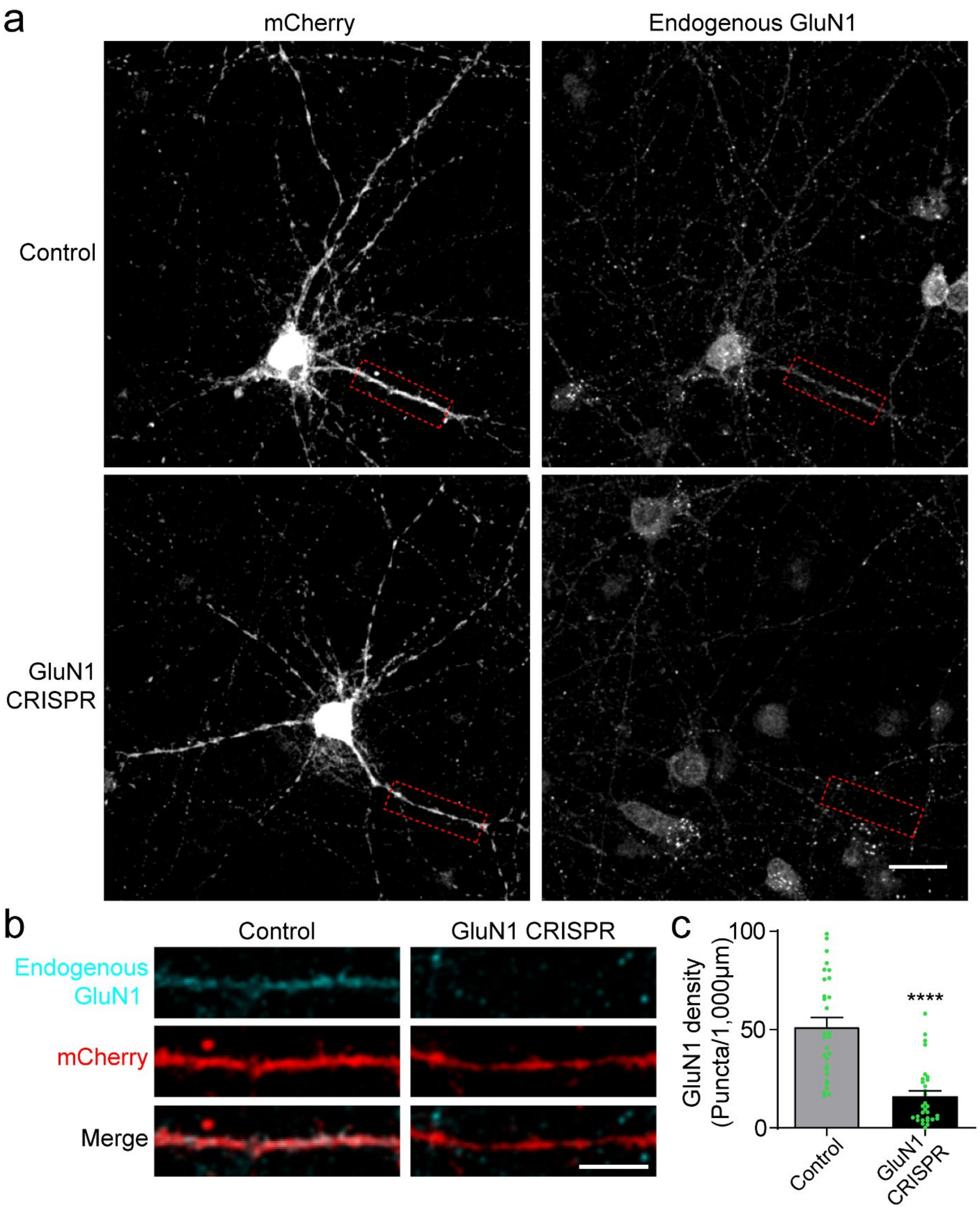

Supplementary Figure 3: a Representative images of DIV 6-9 cortical neurons transfected with GluN1 CRISPR or control vector, together with mCherry to show the morphology. Scale bar = 20μm.

b Zoomed in images of neurons from **a**. Top panels show GluN1 in cyan, middle panels show mCherry fill in red, and bottom panels show merge. Scale bar = 10μm.

c Quantification of endogenous GluN1 puncta density (****p<0.0001, unpaired t-test; green dots represent *n* = 17 cells for each condition).

Supplementary Figure 4: GluN1 hinge region mutants traffic to neuron surface normally.

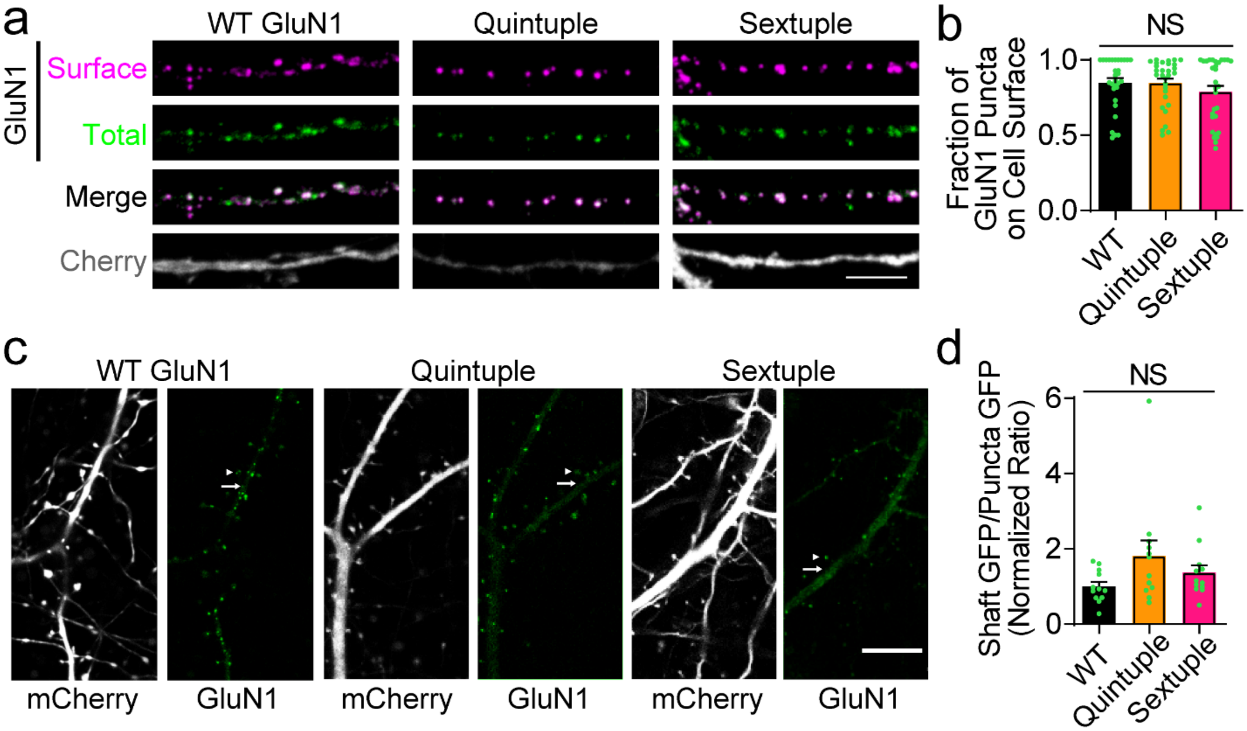

Supplementary Figure 4: **a** Representative images of dendrites of DIV 6-9 cortical neurons transfected with EGFP-GluN1 (WT or mutants as indicated), together with mCherry, GluN2B, and CRISPR constructs to knock out endogenous GluN1. Top panels show surface localized GluN1 (magenta) of neurons live cell-stained for 10 minutes. Cells were then fixed, permeabilized, and stained for total GluN1 (green). Bottom panels show mCherry (white) to show morphology of the dendrites. Scale bar = 5µm.

b Quantification of the fraction of total GluN1 that is on the cell surface. (p=0.3683, ANOVA; green dots represent *n* = 30 cells for each condition).

c Representative images of cortical neurons transfected with either Syn-EGFP-GluN1 WT, Quintuple, or Sextuple (green) together with GluN2B, CRISPR construct targeting

endogenous GluN1, and mCherry (white). Arrow points to dendritic shaft, Arrow Head indicates a dendritic spine. Scale bar:10μm.

d The ratio of average GFP pixel intensities in dendritic shafts to the average GFP intensities per pixel in puncta was used to measure the fraction of diffuse EGFP-GluN1. ($p=0.1089$ WT vs. Quintuple; $p=0.6151$ WT vs. Sextuple; ANOVA; green dots represent n = 12 cells for each condition).

Supplementary Figure 5: EGFP-GluN1 Mutants Flux Calcium.

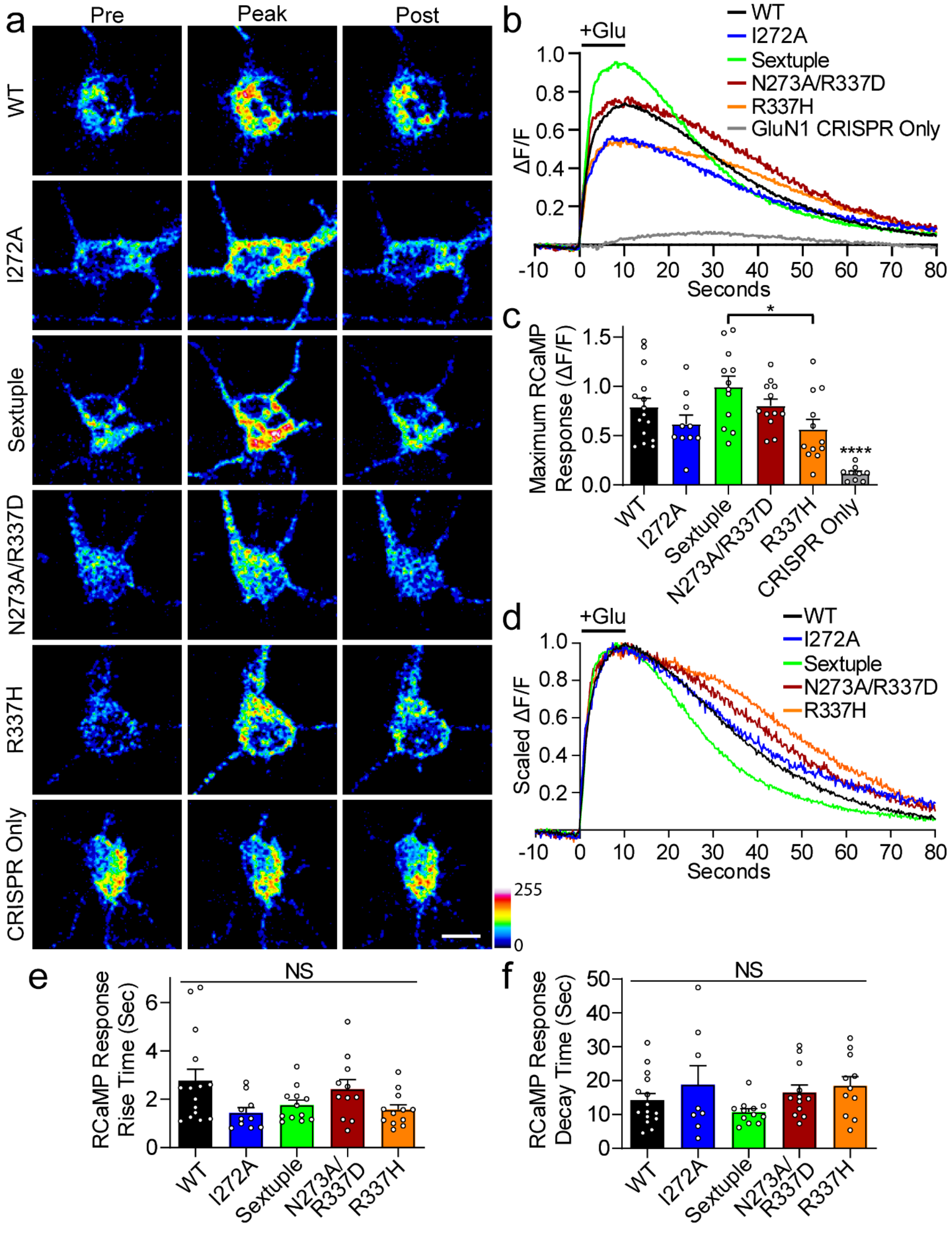

Supplementary Figure 5: a Representative images of DIV 8-10 rat cortical neurons transfected with RCaMP, GluN1 CRISPR, EGFP-GluN1 (WT or mutants as indicated), and GluN2B or with RCaMP and CRISPR alone. Pre images are 10 seconds before glutamate addition, Peak images are the maximum signal during glutamate addition, and Post images are 70 seconds after glutamate was turned off. Scale bar = 10μm.

b Quantification of the response curve of different GluN1 mutants in DIV 8-10 cortical neurons. Graphs represent mean intensity, not normalized or scaled. Glutamate was perfused on cells from t=0 seconds to t=10 seconds as indicated.

c Average peak RCaMP signal related to S5b. (*p=0.0145, Sextuple vs. R337H; ****p<0.0001 WT vs. CRISPR Only; ANOVA; black and white dots represent WT *n* = 15 cells; I272H *n* = 10; Sextuple *n* = 12; N273A/R337D *n* = 12; R337H *n* = 12; CRISPR Only *n* = 9).

d The same response curves as in S5b scaled to a peak of 1.

e Quantification of the average rise time between 10% and 70% of maximal response. (p=0.0309, ANOVA followed by Tukey's; comparisons showed no significant differences; black and white dots represent WT *n* = 15 cells; I272H *n* = 10; Sextuple *n* = 12; N273A/R337D *n* = 11; R337H *n* = 12).

f Quantification of the average decay time between maximal response and 63% of maximal response. (p=0.1836, ANOVA followed by Tukey's; black and white dots represent WT *n* = 15 cells; I272H *n* = 8; Sextuple *n* = 12; N273A/R337D *n* = 12; R337H *n* = 11).

Supplementary Figure 6: Role of Glycosylation.

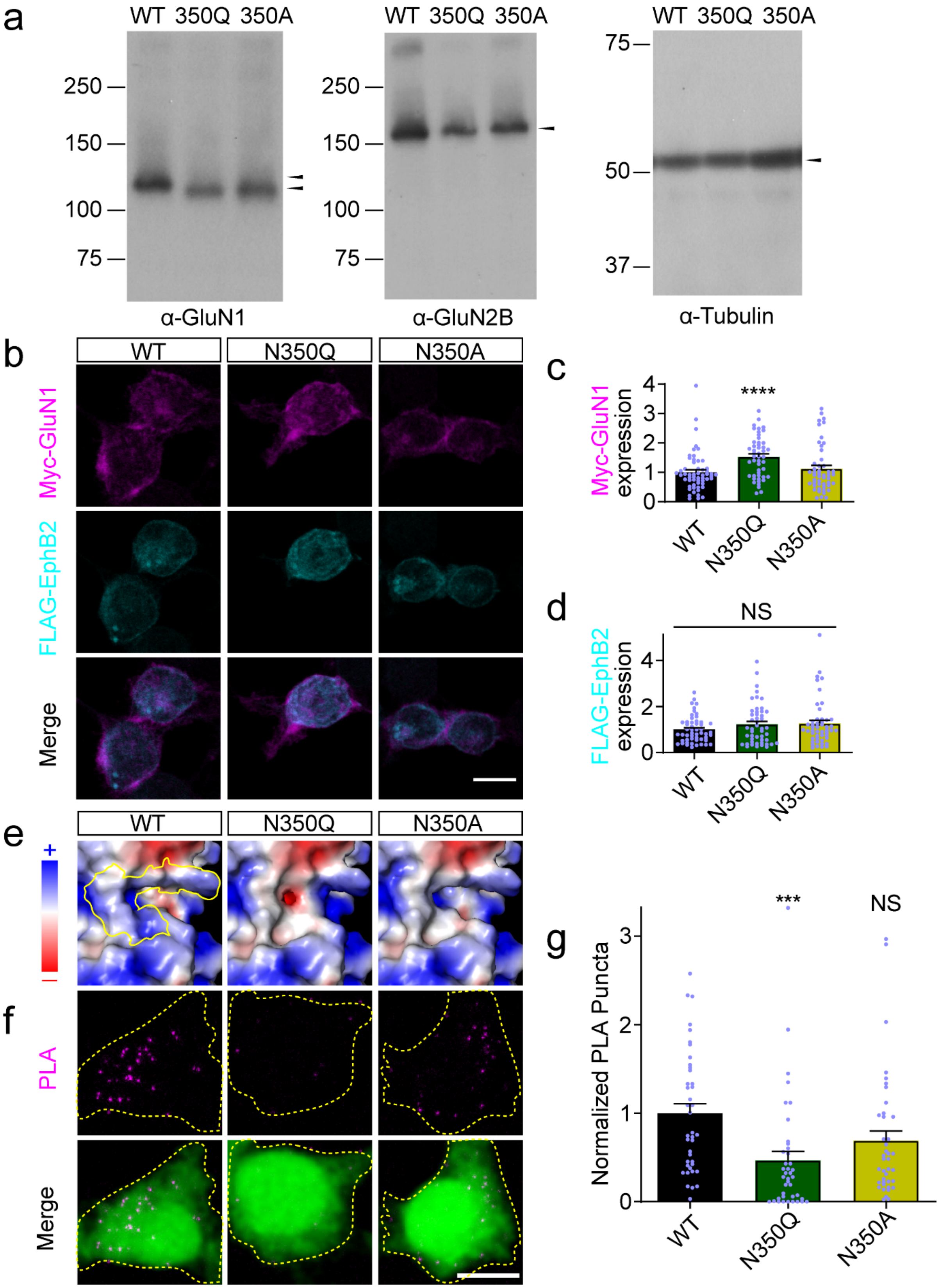

Supplementary Figure 6: a Western blot of lysates from HEK293T cells transfected with GluN1 WT or glycosylation mutants as indicated, and GluN2B. Lysate blots were probed with α-GluN1 (left), α-GluN2B (middle), and α-tubulin as a loading control (right). Arrows indicate appropriate bands. N350 mutation shows a lower apparent molecular weight due to predicted loss of glycosylation compared to GluN1 WT. Complete blots are shown.

b Representative images showing immunocytochemistry of Myc-GluN1 and FLAG-EphB2 expression. Myc-GluN1 mutants are in magenta and FLAG-EphB2 is in cyan. Scale bar = 10μm.

c Quantification of Myc-GluN1 expression level using fluorescent intensity. Values are normalized to the average of WT transfected group. Mutants are not less than WT (****p=0.0009, ANOVA followed by Tukey's; blue dots represent *n* = 30 cells for each condition).

d Quantification of FLAG-EphB2 expression level using fluorescence intensity. Values are normalized to the average of WT transfected group. (p=0.2426, ANOVA; blue dots represent *n* = 30 cells for each condition).

e Surface charge maps of GluN1 NTD hinge region of the indicate GluN1 glycosylation mutants, with blue representing positive charge and red representing negative charge.

f Representative images of PLA results in HEK293T cells. HEK293T cells were transfected with the indicated Myc-GluN1 glycosylation mutants, together with GluN2B, FLAG-EphB2, and EGFP. The upper panels show PLA signal alone. The lower panels are merged images of EGFP in green and PLA signal in magenta. Scale bar = 10μm.

g Quantification of the effects of GluN1 mutants on PLA puncta number. PLA puncta number are quantified by counting the number of puncta per 100 μm^2 in $EGFP^+$ cells. ($p=0.0026$, ANOVA followed by Tukey's; $^{**}p=0.0018$ WT vs. N350Q; $p=0.1055$ WT vs. N350A; $p=0.3078$ N350Q vs. N350A; blue dots represent $n = 40$ cells for each condition).

Supplementary Figure 7: Expression of GluN1 point mutants in HEK293T cells.

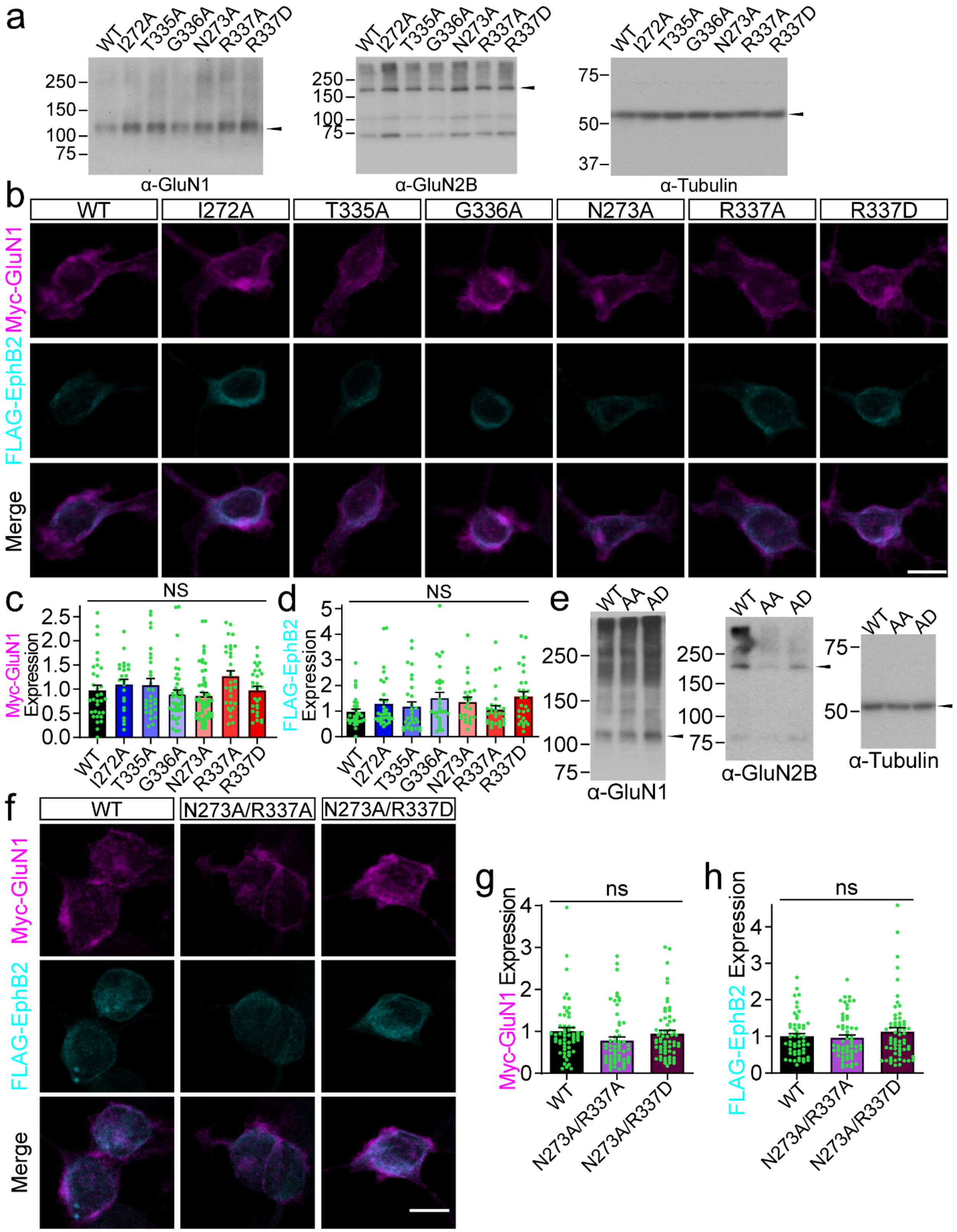

Supplementary Figure 7: a Western blot of lysates from HEK293T cells transfected with GluN1 WT or mutants as indicated, and GluN2B. Lysate blots were probed with α-GluN1 (left), α-GluN2B (middle), and α-tubulin as a loading control (right). Arrows indicate appropriate bands. Single point mutations show no difference in apparent molecular weight. Complete blots are shown.

b Representative images showing immunocytochemistry in non-permeabilized HEK293T cells of Myc-GluN1 and FLAG-EphB2 expression. Myc-GluN1 mutants are in magenta and FLAG-EphB2 is in cyan. Scale bar = 10μm.

c Quantification of Myc-GluN1 expression level using fluorescence intensity. Values are normalized to the average of WT transfected group. ($p=0.0613$, ANOVA; green dots represent WT n = 32 cells; I272A n = 23; T335A n = 29; G336A n = 41; N273A n = 55; R337A n = 30; R337D n = 28).

d Quantification of FLAG-EphB2 expression level using fluorescence intensity. Values are normalized to the average of WT transfected group. ($p=0.1349$, ANOVA; green dots represent WT n = 32 cells; I272A n = 23; T335A n = 30; G336A n = 28; N273A n = 34; R337A n = 30; R337D n = 28).

e Western blot of lysates from HEK293T cells transfected with GluN1 WT or mutants as indicated, and GluN2B. Lysate blots were probed with α-GluN1 (left), α-GluN2B (middle), and α-tubulin as a loading control (right). Arrows indicate appropriate bands. Double point mutations show no difference in apparent molecular weight. Complete blots are shown.

f Representative images showing immunocytochemistry in non-permeabilized HEK293T cells of Myc-GluN1 and FLAG-EphB2 expression. Myc-GluN1 mutants are in magenta and FLAG-EphB2 is in cyan. Scale bar = 10μm.

g Quantification of Myc-GluN1 expression level using fluorescence intensity. Values are normalized to the average of WT transfected group. (p=0.1921, ANOVA; green dots represent WT *n* = 56 cells; N273A/R337A *n* = 58; N273A/R337D *n* = 61).

h Quantification of FLAG-EphB2 expression level using fluorescence intensity. Values are normalized to the average of WT transfected group. (p=0.4097, ANOVA; green dots represent WT *n* = 52 cells; N273A/R337A *n* = 58; N273A/R337D *n* = 61).

Supplementary Figure 8: GluN1 point mutants reduce co-immunoprecipitation with EphB2.

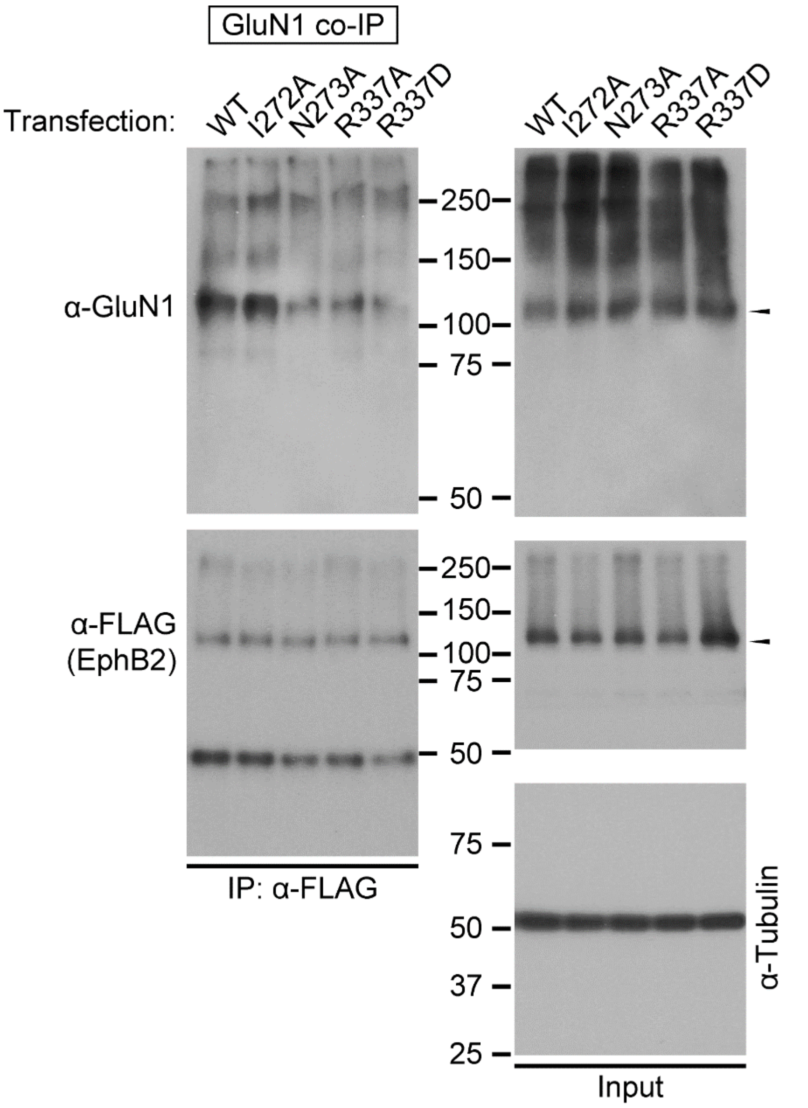

Supplementary Figure 8: Co-immunoprecipitation (co-IP) of GluN1 and EphB2 with α-FLAG (EphB2) antibody from HEK293T cells. Cells are transfected with GluN1 WT or mutants as indicated, GluN2B, and FLAG-EphB2. Left panels are IP samples pulled down with α-FLAG antibody, right panels are lysate inputs. Blots were probed with α-GluN1 (top), α-FLAG (middle), and α-tubulin as a loading control (bottom right). Arrows

indicate appropriate bands. Complete blots are shown. (n = 5 independent experiments).

Supplementary Figure 9: GluN1 point mutants traffic to neuron surface normally but affect dendritic spine density

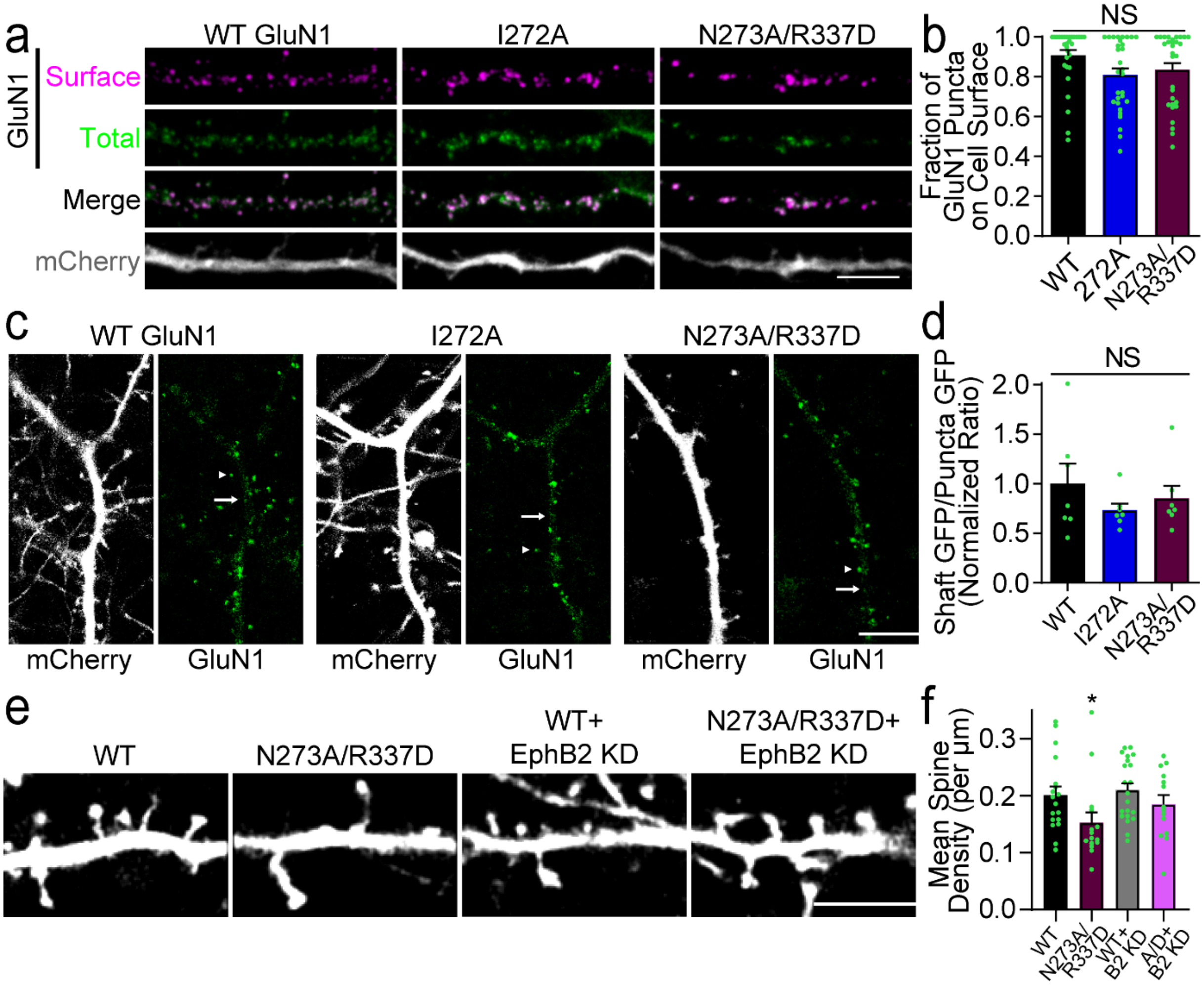

Supplementary Figure 9: **a** Representative images of dendrites of DIV 6-9 cortical neurons transfected with EGFP-GluN1 (WT or mutants as indicated), together with mCherry, GluN2B, and CRISPR constructs to knock out endogenous GluN1. Top panels show surface localized GluN1 (magenta) of neurons live cell-stained for 10 minutes. Cells were then fixed, permeabilized, and stained for total GluN1 (green). Bottom panels show mCherry (white) to show dendritic morphology. Scale bar = 5µm.

b Quantification of the fraction of total GluN1 that is on the cell surface. ($p=0.0747$, ANOVA; green dots represent n = 30 cells for each condition).

c Representative images of DIV 21-23 cortical neurons transfected with either EGFP-GluN1 WT, I272A, or N273A/R337D (green) together with GluN2B, CRISPR construct targeting endogenous GluN1, and mCherry (white). Arrow points to dendritic shaft, Arrow Head indicates a dendritic spine.

d The ratio of average GFP pixel intensities in dendritic shafts to the GFP intensities per pixel in puncta was used to measure the fraction of diffuse EGFP-GluN1. ($p=0.4315$, ANOVA followed by Tukey's; $p=0.4003$ WT vs. I272A; $p=0.7447$ WT vs. AD; green dots represent n = 7 cells for each condition).

e Representative images of dendrites of DIV 21-23 cortical neurons transfected with EGFP-GluN1 (WT or N273A/R337D), together with mCherry, GluN2B, and CRISPR constructs to knock out endogenous GluN1, with and without EphB2 knockdown via RNAi.

f Quantification of effects of GluN1 point mutants on dendritic spine density (*$p=0.0392$, ANOVA followed by Tukey's; green dots represent WT n = 18 cells; N273A/R337D n = 15; B2 knockdown+WT n = 21; B2 knockdown+N273A/R337D n = 14).

Supplementary Figure 10: pH sensitive histidine mutants traffic to cell surface normally.

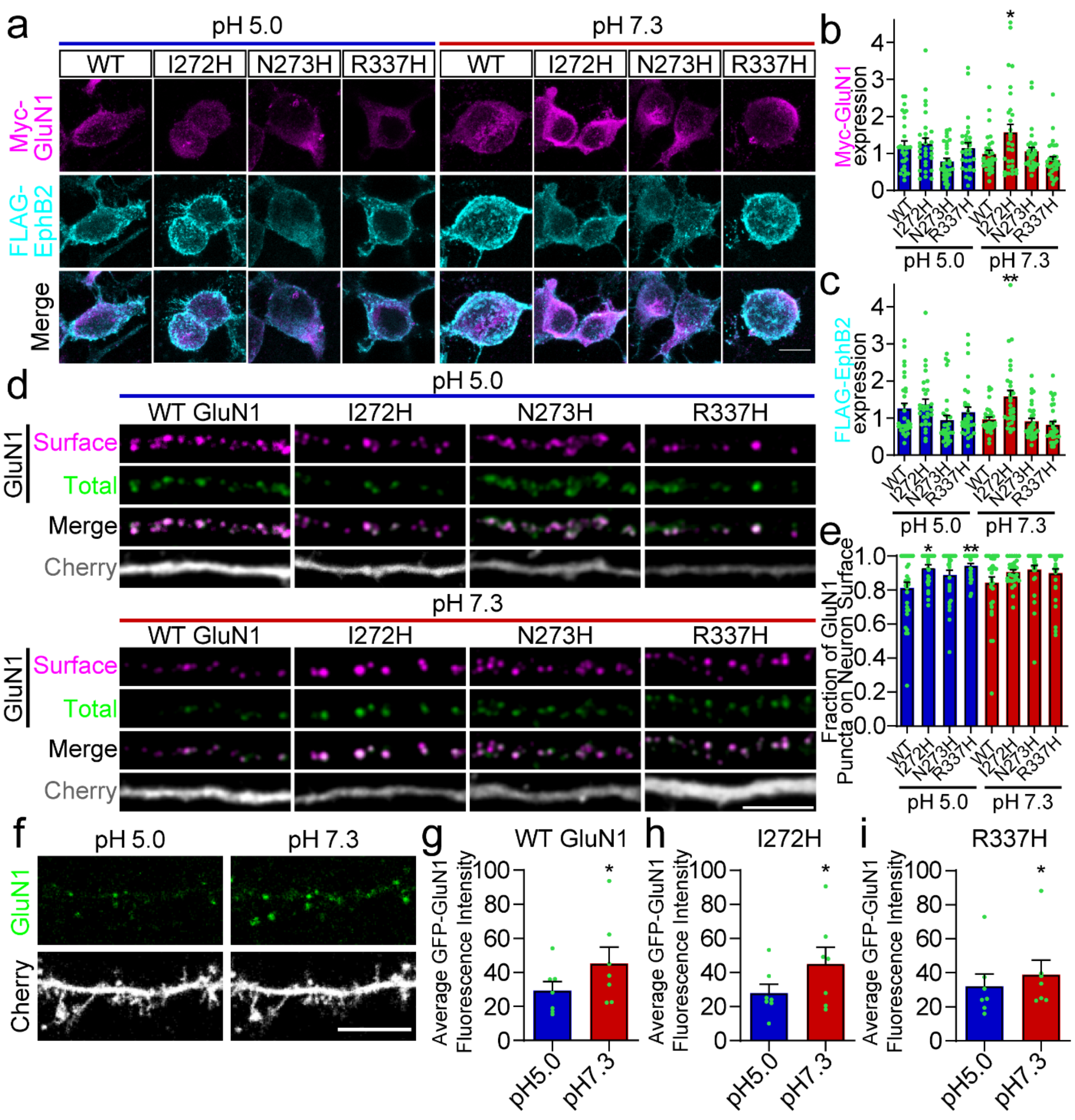

Supplementary Figure 10: a Representative images showing immunocytochemistry in non-permeabilized HEK293T cells of Myc-GluN1 and FLAG-EphB2 expression. Cells

were treated with media of the indicated pH for 30 minutes prior to fixation. Myc-GluN1 mutants are in magenta and FLAG-EphB2 is in cyan. Scale bar = 10μm.

b Quantification of Myc-GluN1 expression level using fluorescence intensity. Values are normalized to the average of WT-pH7.3 transfected group. Mutants are not less than WT (*p=0.0341 WT-pH7.3 vs. I272H-pH7.3; ANOVA; green dots represent *n* = 30 cells for each condition). Error bars show S.E.M.

c Quantification of FLAG-EphB2 expression level using fluorescence intensity. Values are normalized to the average of WT-pH7.3 transfected group. Mutants are not less than WT (**p=0.0090 WT-pH7.3 vs. I272H-pH7.3; ANOVA; green dots represent *n* = 30 cells for each condition). Error bars show S.E.M.

d Representative images of dendrites of DIV 6-9 cortical neurons transfected with EGFP-GluN1 (WT or mutants as indicated), together with mCherry, GluN2B, and CRISPR constructs to knock out endogenous GluN1. Cells were treated with media at either pH 5.0 or pH 7.3 for 30 min before staining. Top panels show surface localized GluN1 (magenta) of neurons live cell-stained for 10 minutes in their respective pH adjusted medias. Cells were then fixed, permeabilized, and stained for total GluN1 (green). Bottom panels show mCherry (white) to show morphology of the dendrites. Scale bar = 5μm.

e Quantification of the fraction of total GluN1 that is on the cell surface. Mutants are not less than WT (*p=0.0185 WT-pH5.0 vs. I272H-pH5.0; **p=0.0065 WT-pH5.0 vs. R337H-pH5.0; ANOVA; green dots represent WT-pH5.0 *n* = 30 cells; I272H-pH5.0 *n* = 30; N273H-pH5.0 *n* = 27; R337H-pH5.0 *n* = 30; WT-pH7.3 *n* = 30; I272H-pH7.3 *n* = 30; N273H-pH7.3 *n* = 30; R337H-pH7.3 *n* = 30). Error bars show S.E.M.

f Representative images of the effects of pH on EGFP-GluN1 fluorescence in dendrites of DIV 21-23 cortical neurons transfected with EGFP-GluN1 WT together with mCherry, GluN2B, and CRISPR constructs to knock out endogenous GluN1. Cells were treated with media at either pH 5.0 or pH 7.3 as indicated for less than 5 min before imaging.
g Quantification of the average EGFP fluorescence intensity of WT GluN1 transfected neurons at pH 5.0 and pH 7.3. The same cell was imaged for each comparison. (*p=0.0182, paired t-test; green dots represent *n* = 7 cells). Error bars show S.E.M.
h Quantification of the average EGFP fluorescence intensity of I272H GluN1 transfected neurons at pH 5.0 and pH 7.3. The same cell was imaged for each comparison. (*p=0.0209, paired t-test; green dots represent *n* = 7 cells). Error bars show S.E.M.
i Quantification of the average EGFP fluorescence intensity of R337H GluN1 transfected neurons at pH 5.0 and pH 7.3. The same cell was imaged for each comparison. (*p=0.0366, paired t-test; green dots represent *n* = 7 cells). Error bars show S.E.M.

Primers

GluN1_I272A_F:	GACTTCAGCTCGCCAATGGC
GluN1_I272A_R:	GCCATTGGCGAGCTGAAGTC
GluN1_N273A_F:	CTTCAGCTCATCGCTGGCAAG
GluN1_N273A_R:	CTTGCCAGCGATGAGCTGAAG
GluN1_T335A_F:	GGAGTGGCTGGCCGTG
GluN1_T335A_R:	CACGGCCAGCCACTCC
GluN1_G336A_F:	GAGTGACTGCCCGTGTGG
GluN1_G336A_R:	CCACACGGGCAGTCACTC
GluN1_R337A_F:	GAGTGACTGGCGCTGTGGAATT
GluN1_R337A_R:	AATTCCACAGCGCCAGTCACTC
GluN1_R337D_F:	GTGACTGGCGATGTGGAATTC
GluN1_R337D_R:	GAATTCCACATCGCCAGTCAC
GluN1_N350A_F:	GGAAGTTTGCCGCCTATAGTATCATG
GluN1_N350A_R:	CATGATACTATAGGCGGCAAACTTCC
GluN1_N350Q_F:	GGAAGTTTGCCCAATATAGTATCATG
GluN1_N350Q_R:	CATGATACTATATTGGGCAAACTTCC
GluN1_I272H_F:	TCGGACTTCAGCTCCACAATGGCAAGAATGA
GluN1_I272H_R:	TCATTCTTGCCATTGTGGAGCTGAAGTCCGA
GluN1_N273H_F:	GGACTTCAGCTCATCCATGGCAAGAATGAGTCA
GluN1_N273H_R:	TGACTCATTCTTGCCATGGATGAGCTGAAGTCC
GluN1_R337H_F:	ACGGAGTGACTGGCCATGTGGAATTCAATGA
GluN1_R337H_R:	TCATTGAATTCCACATGGCCAGTCACTCCGT
