# Supplementary material for: Positive surface charge of GluN1 N-terminus mediates the direct interaction with EphB2 and NMDAR mobility
Source: Nat Commun. 2020 Jan 29;11:570. doi: 10.1038/s41467-020-14345-6 (PMC6989673; doi:10.1038/s41467-020-14345-6)
Supplement: Supplementary file 2 — Reporting Summary [file 41467_2020_14345_MOESM2_ESM.pdf]

nature research

Corresponding author(s): DBPR NCOMMS-19-00878

Last updated by author(s): Dec 1, 2019

Reporting Summary

Nature Research wishes to improve the reproducibility of the work that we publish. This form provides structure for consistency and transparency in reporting. For further information on Nature Research policies, see Authors & Referees and the Editorial Policy Checklist .

Statistics

For all statistical analyses, confirm that the following items are present in the figure legend, table legend, main text, or Methods section.

n/a	Confirmed	
☐	☒	The exact sample size (n) for each experimental group/condition, given as a discrete number and unit of measurement
☐	☒	A statement on whether measurements were taken from distinct samples or whether the same sample was measured repeatedly
☐	☒	The statistical test(s) used AND whether they are one- or two-sided<br>*Only common tests should be described solely by name; describe more complex techniques in the Methods section.*
☒	☐	A description of all covariates tested
☐	☒	A description of any assumptions or corrections, such as tests of normality and adjustment for multiple comparisons
☐	☒	A full description of the statistical parameters including central tendency (e.g. means) or other basic estimates (e.g. regression coefficient) AND variation (e.g. standard deviation) or associated estimates of uncertainty (e.g. confidence intervals)
☐	☒	For null hypothesis testing, the test statistic (e.g. F, t, r) with confidence intervals, effect sizes, degrees of freedom and P value noted<br>*Give P values as exact values whenever suitable.*
☒	☐	For Bayesian analysis, information on the choice of priors and Markov chain Monte Carlo settings
☒	☐	For hierarchical and complex designs, identification of the appropriate level for tests and full reporting of outcomes
☒	☐	Estimates of effect sizes (e.g. Cohen's d, Pearson's r), indicating how they were calculated

Our web collection on statistics for biologists contains articles on many of the points above.

Software and code

Policy information about availability of computer code

Data collection	Leica LAS X Imaging Software was used to collect confocal and FRAP images.
Data analysis	ImageJ was used to analyze data, GraphPad Prism was used for statistical analysis, Adaptive Poisson-Boltzmann Solver plugin in PyMol was used to determine surface potential of published crystal structures.

For manuscripts utilizing custom algorithms or software that are central to the research but not yet described in published literature, software must be made available to editors/reviewers. We strongly encourage code deposition in a community repository (e.g. GitHub). See the Nature Research guidelines for submitting code & software for further information.

Data

Policy information about availability of data

All manuscripts must include a data availability statement. This statement should provide the following information, where applicable:

- Accession codes, unique identifiers, or web links for publicly available datasets
- A list of figures that have associated raw data
- A description of any restrictions on data availability

Provide your data availability statement here.

Field-specific reporting

Please select the one below that is the best fit for your research. If you are not sure, read the appropriate sections before making your selection.

☒ Life sciences ☐ Behavioural & social sciences ☐ Ecological, evolutionary & environmental sciences

Life sciences study design

All studies must disclose on these points even when the disclosure is negative.

Sample size	Sample sizes were determined so that experiments be replicated to ensure confidence in the results and were based on previous studies (Dalva et al., 2000, Kayser et al., 2008, Nolt et al., 2011, and Hruska et al., 2015). For all experiments there was enough statistical power to detect the corresponding effect size.
Data exclusions	All data acquired were included in analyses.
Replication	Each experiment was replicated a minimum of three times and data was reliably reproduced with each replication attempt.
Randomization	Data were acquired and analyzed based on the standards in the field, however, no method of randomization was used to determine how sample were allocated to experimental groups and processed.
Blinding	Data were collected blind to condition and analyzed blind to condition.

Reporting for specific materials, systems and methods

We require information from authors about some types of materials, experimental systems and methods used in many studies. Here, indicate whether each material, system or method listed is relevant to your study. If you are not sure if a list item applies to your research, read the appropriate section before selecting a response.

Materials & experimental systems

n/a	Involved in the study	
☐	☒	Antibodies
☐	☒	Eukaryotic cell lines
☒	☐	Palaeontology
☐	☒	Animals and other organisms
☒	☐	Human research participants
☒	☐	Clinical data

Methods

n/a	Involved in the study	
☒	☐	ChIP-seq
☒	☐	Flow cytometry
☒	☐	MRI-based neuroimaging

Antibodies

Antibodies used

Primary antibodies:
mouse monoclonal (IgG1) anti-GluN1 (1:500, BioLegend, clone R1JHL, cat# 828201, lot# B212895)
goat polyclonal anti-EphB2 (1:1200 (ICC), R&D Systems, cat# AF467, lot# CVT0315041)
mouse monoclonal (IgG1) anti-EphB2 (1: 500 (WB), Invitrogen, clone 1A6C9, cat# 37-1700, lot# RD215698)
rabbit polyclonal anti-Myc (1:3000, Abcam, Cambridge, MA, cat# ab9103, lot# 2932489)
mouse monoclonal (IgG2b) anti-GluN2B (1:500, Neuromab, UC Davis, Davis, CA, clone N59/36, cat# 75-101, lot# 455-10JD-82)
mouse monoclonal (IgG2A) anti-PSD-95 (1:2500, Neuromab, UC Davis, Davis, CA, clone 28/43, cat# 75-028, lot# 455.7JD.22f)
mouse monoclonal (IgG1) anti-Synaptophysin-1 (1:5000, Synaptic Systems, Gottingen, Germany, clone 7.2, cat# 101 111, lot# 101011/1-43)
guinea pig polyclonal anti-vesicular glutamate transporter 1 (vGlut1; 1:5000, EMD Millipore, Temecula, CA, cat# AB5905, lot# 2932489)
rabbit polyclonal anti-GFP (1:3000, Life Technologies, cat# A6455, lot# 1736965)
mouse monoclonal (IgG1) anti-GAPDH (1:500, EMD Millipore, Temecula, CA, cat# MAB374, lot# 2910381)
rabbit polyclonal anti-Tubulin (1:10,000, Abcam, Cambridge, MA, cat# ab18251, lot# GR235480-2)
rabbit polyclonal anti-actin (1:2000, Sigma, cat# A2103, lot# 115M4865V)
rabbit polyclonal anti-FLAG (1:1000, Sigma, Cat# F7425)
Secondary antibodies:
Donkey anti-mouse-HRP (1:10,000, Jackson ImmunoResearch, cat# 715-035-151, lot# 128396)
Donkey anti-rabbit-HRP (1:10,000, Jackson ImmunoResearch, cat# 711-035-152, lot# 132960)
Donkey anti-goat-HRP (1:10,000, Jackson ImmunoResearch, cat# 705-035-147, lot# 112876)
Donkey anti-rabbit unconjugated (1:100, Jackson ImmunoResearch, cat# 711-005-152 lot# 125861)
Donkey anti-mouse AlexaFluor-488 (1:500, Jackson ImmunoResearch, cat# 715-545-150, lot# 11603)
Donkey anti-rabbit AlexaFluor-488 (1:500, Jackson ImmunoResearch, cat# 711-545-152, lot# 126601)
Donkey anti-goat Cy3(1:500, Jackson ImmunoResearch, cat# 705-166-147, lot# 107019)
Donkey anti-rabbit Cy3 (1:500, Jackson ImmunoResearch, cat# 711-165-152, lot# 123091)
Donkey anti-guinea pig AlexaFluor-647 (1:500, Jackson ImmunoResearch, cat# 706-605-148, lot# 116734)
Donkey anti-goat AlexaFluor-647 (1:500, Jackson ImmunoResearch, cat# 706-606-147, lot# 124186)

Validation	All primary and secondary antibodies were profiled in our previous publications and published work from other labs and were reported to be specific (Kayser et al., 2008, McClelland et al., 2010, Nolt et al., 2011, Hruska, et al, 2015 and Hanamura et al, 2017).

Eukaryotic cell lines

Policy information about cell lines

Cell line source(s)	HEK293T Cells - source: ATCC
Authentication	HEK293T cells were used in our previous publications and published work from other labs (Dalva et al., 2000 and Hanamura et al, 2017).
Mycoplasma contamination	Cell lines were not tested for mycoplasma contamination.
Commonly misidentified lines (See ICLAC register)	N/A

Animals and other organisms

Policy information about studies involving animals; ARRIVE guidelines recommended for reporting animal research

Laboratory animals	P21 mice for whole brain synaptosome experiments were obtained from timed pregnant CD-1 mice purchased from Charles River Laboratories Inc. (Wilmington, MA) and housed (3-5 mice per cage) in Thomas Jefferson University's laboratory animal facility. Standard housing was used with normal light/dark cycle. E17-18 rat embryos from timed pregnant animals purchased from Charles River Laboratories Inc. (Wilmington, MA) were used to make primary cortical neuron cultures . Both male and female mouse and rat pups were used for the experiments.
Wild animals	This study did not involve wild animals.
Field-collected samples	This study did not involve samples collected from the field.
Ethics oversight	All animal studies were approved by the Institutional Animal Care and Use Committee guidelines at Thomas Jefferson University in accordance with US National Institutes of Health guidelines.

Note that full information on the approval of the study protocol must also be provided in the manuscript.
